# Ascorbate Recycling as a Molecular Redox Capacitor: A Sulfur-Centered Perspective on Dehydroascorbate Reduction in Biological Systems

**DOI:** 10.3390/cells15151391

**Published:** 2026-07-31

**Authors:** Rika Heshiki, Kakeru B. Mizumoto, Riko F. Naomasa, Takashi Matsumura, Hideo Yamasaki

**Affiliations:** Faculty of Science, University of the Ryukyus, Nishihara, Okinawa 903-0213, Japan

**Keywords:** Ascorbic acid (AsA), dehydroascorbic acid (DHA), hydrogen sulfide (H_2_S), monodehydroascorbic acid (MDHA), neutrophils, oxidative stress, persulfides, photosynthesis, polysulfides, reactive oxygen species (ROS), reactive sulfur species (RSS)

## Abstract

**Highlights:**

**What are the main findings?**

- Ascorbate recycling can be viewed as a multilayered system comprising nonenzymatic chemistry, enzymatic acceleration, spatial organization, and integration with broader redox and nutritional networks.
- Sulfur chemistry repeatedly appears across DHA-reducing systems, including GSH, thiol enzymes, hydrogen sulfide (H_2_S), and possibly reactive sulfur species (RSS), although RSS-dependent DHA reduction remains a chemically plausible but experimentally unresolved possibility.

**What are the implications of the main findings?**

- The AsA/DHA couple can be conceptualized as a molecular redox capacitor that stores, releases, and regenerates reducing capacity during fluctuating oxidative stress.
- Recent findings on moonlighting proteins and extracellular AsA–GSH recycling systems suggest that ascorbate recycling is not merely a soluble antioxidant reaction, but also a spatially organized redox–membrane and compartmental buffering system.

**Abstract:**

Ascorbate (AsA), or vitamin C, is a central redox metabolite that functions as an antioxidant, enzyme cofactor, and electron donor. Its cellular function depends not only on biosynthesis or dietary uptake, but also on rapid recycling from its oxidized forms, monodehydroascorbate (MDHA) and dehydroascorbate (DHA). This requirement is especially evident in high-demand systems such as plant chloroplasts, which face continuous photosynthetic reactive oxygen species (ROS) production under illumination, and human neutrophils, which accumulate millimolar ascorbate to withstand NADPH oxidase-driven oxidative bursts in pathogen defense. Here, we revisit ascorbate recycling from a sulfur-centered perspective. Historical studies of plant, animal, and solution-chemistry pathways show that many DHA-reducing systems converge on sulfur chemistry, including glutathione (GSH), cysteine-dependent enzymes, H_2_S, and modified thiols. We propose that ascorbate recycling is organized as a multilayered system in which nonenzymatic reactions are accelerated by enzymes, localized within cellular or extracellular compartments, and integrated with broader NAD(P)H-, glutathione-, sulfur-, and diet-dependent redox networks. Within this framework, the AsA/DHA couple can be viewed as a molecular redox capacitor that buffers transient oxidative pressure. Reactive sulfur species (RSS), including persulfides and polysulfides, represent chemically plausible but experimentally unresolved contributors to DHA reduction.

## 1. Introduction

### 1.1. Non-Uniform Distribution of Ascorbate in Humans and Plants

Humans and several animal lineages cannot synthesize L-ascorbic acid (AsA), commonly referred to as ascorbate or vitamin C, because they have lost functional L-gulono-γ-lactone oxidase (GULO), the terminal enzyme in the animal ascorbate biosynthetic pathway (Figure 1). In this terminal reaction, GULO oxidizes L-gulono-γ-lactone to ascorbate in the presence of molecular oxygen (O_2_), generating hydrogen peroxide (H_2_O_2_) as a canonical byproduct [1,2]. Because H_2_O_2_ is a reactive oxygen species (ROS) that potentially causes oxidative damage to cells, loss of GULO function may have been evolutionarily advantageous by reducing endogenous ROS generation [3,4]. Animals that have lost the capacity for ascorbate biosynthesis therefore depend on dietary sources of ascorbate. In humans, plant-derived foods, particularly fresh fruits and vegetables, are the major dietary sources of vitamin C [5,6].

Although ascorbate is one of the most abundant low-molecular-weight metabolites in both animals and plants [3,11,12], its distribution across tissues, organs, and subcellular compartments is strikingly non-uniform (Figure 2). In humans, reported ascorbate concentrations vary widely among tissues and body fluids, ranging from low micromolar levels in plasma to substantially higher, often millimolar, levels in organs and tissues such as the pituitary, adrenal glands, ocular lens, and brain [12,13]. A similarly steep gradient is observed in vascular plants, where ascorbate levels are generally lower in roots and higher in photosynthetically active green leaves [14,15,16]. Glutathione (GSH) is likewise maintained at millimolar concentrations in many animal and plant cells [17,18,19,20].

**Figure 3:**
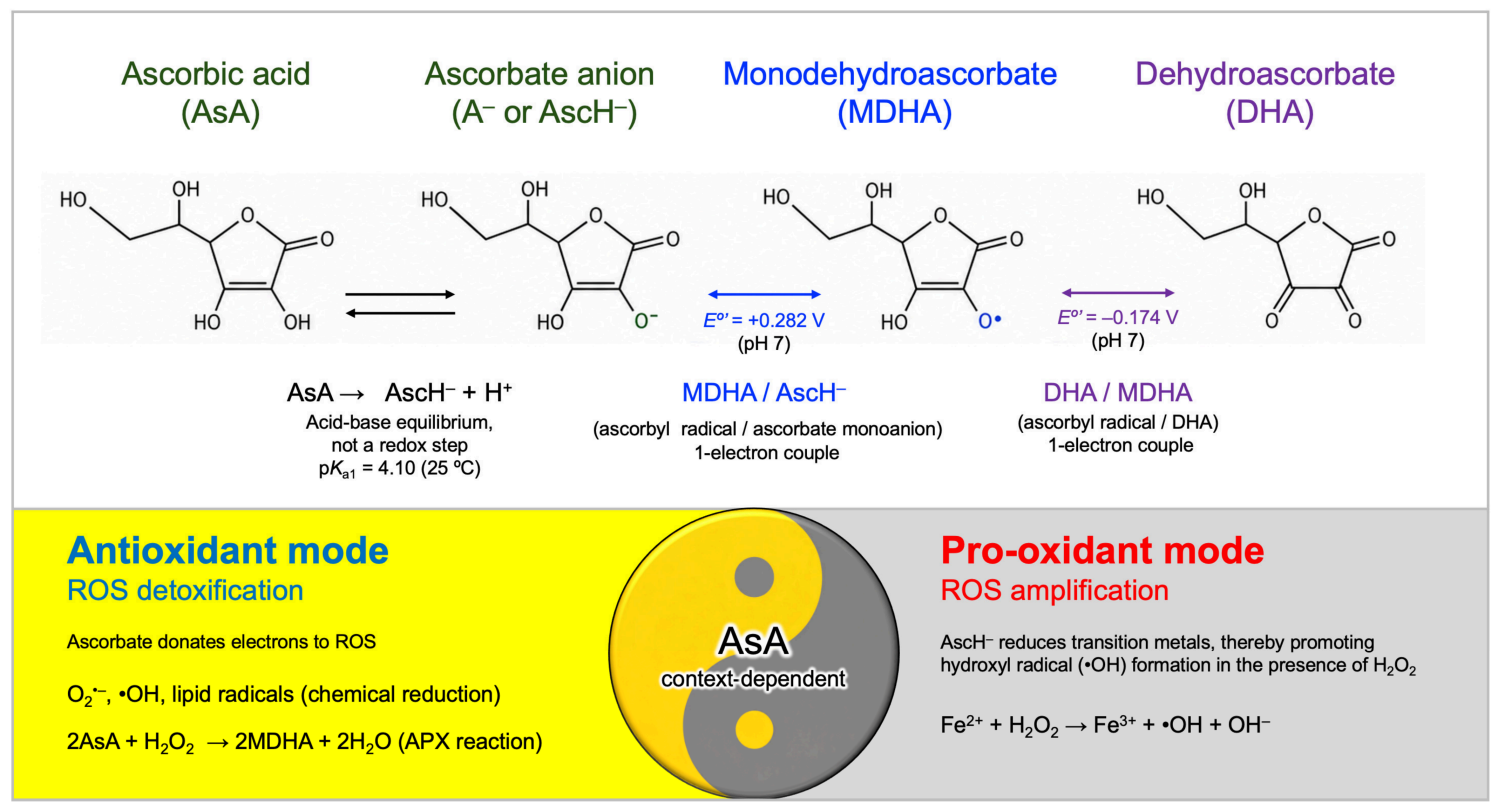
Redox chemistry of ascorbate and its context-dependent antioxidant and pro-oxidant functions. (**Upper panel**) Ascorbic acid (AsA) and the ascorbate monoanion (AscH^−^) interconvert by an acid–base equilibrium, not a redox step. AscH^−^ is oxidized in two one-electron steps via the monodehydroascorbate (MDHA, ascorbyl) radical to dehydroascorbate (DHA). The negative MDHA/DHA potential reflects the instability of DHA, the substrate that recycling systems reduce back to AscH^−^ [32]. (**Lower panel**) The same redox versatility makes ascorbate’s net effect context-dependent (Yin–Yang symbol) [3,6,21]. In antioxidant mode, ascorbate scavenges ROS (O_2_^•−^, •OH, lipid radicals) [33] and donates electrons in the ascorbate peroxidase reaction (APX) [34]. In pro-oxidant mode, AscH^−^ reduces transition metals (Fe^3+^ → Fe^2+^), driving Fenton-type •OH formation [35].

Local ascorbate concentrations appear to track the intensity of ascorbate-dependent biochemistry, in which ascorbate acts both as an antioxidant (Figure 3) and as a specific enzyme cofactor or electron donor [21,22]. In humans, the adrenal glands and pituitary maintain high ascorbate concentrations because ascorbate serves as an electron donor for copper-dependent monooxygenases, including dopamine β-hydroxylase (DBH) [23] and peptidylglycine α-amidating monooxygenase (PAM) [24], which are involved in catecholamine and peptide-hormone biosynthesis, respectively. The brain retains millimolar ascorbate even during dietary depletion, consistent with its roles in neuroprotection [25], neuromodulation [26], and catecholamine synthesis [27].

Neutrophils, key immune cells in pathogen defense [28], accumulate ascorbate to levels 50- to 100-fold higher than those in plasma [29,30]. This enrichment is thought to protect neutrophils against their own oxidative, or respiratory, burst and to support antimicrobial function [28]. In plants, chloroplasts represent a major intracellular ascorbate pool because the photosynthetic apparatus is a continuous source of ROS [31]. Young, proliferating organs, such as flower buds and young leaves, are also rich in ascorbate, reflecting the demand for cell division and for ascorbate-dependent 2-oxoglutarate dioxygenases [3,10].

Despite the evolutionary divergence between animals and plants, these distribution patterns may reflect a shared organizing principle: large ascorbate pools tend to occur at sites of intense ascorbate-dependent metabolism and/or oxidative challenge.

### 1.2. Requirement of Ascorbate Regenerating Mechanisms

In high-demand ascorbate systems, such as neutrophils and photosynthetic cells, the ascorbate pool cannot be maintained by slow de novo synthesis or uptake alone [36,37]. Under conditions of intense oxidative metabolism, ascorbate would be rapidly depleted without efficient recycling mechanisms [31,38]. Thus, the steady-state ascorbate pool reflects not only biosynthesis and uptake but also the capacity to regenerate ascorbate from its oxidation products, monodehydroascorbate (MDHA) and dehydroascorbate (DHA) as illustrated in Figure 4.

In 1951, Stanley Levenson, Hyman Rosen, and George Hitchings reported that DHA could be reduced back to ascorbate in the presence of hydrogen sulfide (H_2_S) [43]. Their study, “Kinetics of the reactions of dehydroascorbic acid in the presence of hydrogen sulfide,” was published long before the modern concepts of H_2_S signaling and reactive sulfur species (RSS) had emerged [44,45,46]. Nevertheless, this early observation of H_2_S-dependent DHA reduction has remained largely outside the mainstream history of redox biology (Figure 5).

At that time, the reaction was interpreted mainly as a problem in vitamin C chemistry, analytical methodology, and chemical kinetics, rather than as a potential clue to physiological ascorbate regeneration [43]. In subsequent decades, DHA reduction was instead incorporated into the framework of glutathione (GSH)-dependent and enzyme-mediated recycling systems. In plant cells, the ascorbate–glutathione cycle, also known as the Foyer–Halliwell–Asada pathway [47], became established as a central recycling mechanism [11,48]. In animal cells, glutaredoxin (Grx)- and thioredoxin (Trx)-related systems, together with other candidate enzymes (including protein disulfide isomerase and 3α-hydroxysteroid dehydrogenase), have been proposed to serve analogous roles in DHA reduction [3,22].

However, because multiple chemical and enzymatic reductants can reduce DHA to regenerate ascorbate, the in vivo molecular machinery and the possible contribution of small chemical reductants, including H_2_S, remain incompletely understood in both plants [47] and animals [22,49]. Recent evidence that GSH, related low-molecular-weight thiols, and protein cysteine residues can undergo persulfidation (-SSH/-SS_n_H) or polysulfidation (-SS_n_-) [50,51] raises the possibility that RSS-linked sulfur chemistry may contribute to DHA reduction and thereby reshape the framework of ascorbate recycling.

This review revisits ascorbate regeneration from a sulfur-centered perspective. We examine the historical development of DHA reduction research, the partly independent progress of plant and animal studies, the changing interpretation of “DHA reductase activity,” and the emergence of “moonlighting enzymes” as contributors to ascorbate recycling. By drawing an analogy between ascorbate redox networks and electrical circuits, we propose that ascorbate recycling can be viewed as a molecular redox capacitor that buffers transient ROS bursts and protects biomolecules from oxidative damage. This perspective may provide a useful conceptual framework for understanding stress adaptation, the possible nutraceutical roles of dietary sulfur compounds, immune-cell redox defense, and redox-associated pathologies, including neurodegenerative diseases and cancer.

## 2. A 100-Year Historical Perspective on DHA Reduction

Although the ascorbate-recycling mechanisms described above can now be viewed as components of a unified conceptual framework, they were not discovered as a coherent scheme. Rather, they emerged piecemeal over nearly 100 years through studies that often developed in separate research communities with limited interaction. This history can be divided into three largely independent lineages—one in plants, one in animals, and one in solution chemistry—that advanced in parallel and have only recently begun to converge (Figure 5). Despite their different starting points, all three lineages ultimately led to the same unresolved question: what reduces DHA in vivo?

### 2.1. An Integrated Origin

#### 2.1.1. Szent-Györgyi’s Early Notion

Albert Szent-Györgyi, who discovered hexuronic acid, later identified as vitamin C, was awarded the 1937 Nobel Prize in Physiology or Medicine for his discoveries in biological oxidation, with special reference to vitamin C. Even before this recognition, he had provided a remarkably prescient framework for ascorbate redox biology. In his 1928 study on peroxidase systems and the chemistry of the adrenal cortex, Szent-Györgyi recognized that “hexuronic acid” was a reversibly oxidizable reducing compound closely linked to biological oxidation–reduction systems [52]. In plant extracts, he showed that added H_2_O_2_ was first consumed by a rapidly reacting “reducing factor” before peroxidase-dependent oxidation of classical substrates became detectable. He further concluded that this factor functioned as a catalytic hydrogen carrier between peroxidase and other oxidizing or reducing systems, rather than as a terminal sacrificial reductant [52].

From the perspective of the present review, two aspects of this work are especially relevant. First, Szent-Györgyi showed that the oxidized reducing factor could be quantitatively reduced again by H_2_S, thereby demonstrating a reversible connection between peroxide-dependent oxidation and sulfur-based chemical reduction.

Notably, Szent-Györgyi already distinguished between two distinct modes of oxidation: hexuronic acid oxidized by iodine or by peroxide/peroxidase could be quantitatively re-reduced by H_2_S, whereas the product formed by autoxidation with molecular oxygen could not be reduced by the same agent [52]. This early mechanistic distinction anticipates the modern contrast between the reversible DHA/ascorbate couple and the irreversible hydrolysis product 2,3-diketogulonate (Figure 4). Second, he recognized that oxidized hexuronic acid could be reduced by animal tissue systems, especially the “Hopkins glutathione system.”

He explicitly raised the unresolved question of whether the unknown factor that reduces disulfide bonds also directly reduces the oxidized acid, or instead acts through labile and fixed –SH groups [52]. By formulating this unresolved question so clearly, Szent-Györgyi exemplified the kind of “problem finder” later emphasized by Hans Selye [44]. This question anticipates, in an early form, the modern problem of how oxidized ascorbate species are reduced in vivo.

#### 2.1.2. Conceptual Prototypes of DHA Reduction Mechanisms

A decade later, Hopkins and Morgan (1936) provided a quantitative kinetic foundation for this field [53]. In the presence of ascorbate oxidase, glutathione (GSH) protected ascorbate from oxidation while being consumed at the same rate at which ascorbate alone would otherwise have been oxidized [53]. This stoichiometric coupling can be viewed, in retrospect, as a conceptual prototype of the ascorbate–glutathione cycle.

By 1951, Mapson and Goddard [54], working with pea, and Conn and Vennesland [55], working with wheat germ, had independently identified an NADPH-dependent enzyme that Mapson and Goddard explicitly termed “glutathione reductase” [54]. Mapson and Goddard also assembled the full sequence—isocitrate or malate → NADPH → GSSG/GSH → DHA/ascorbate—as a hydrogen-transfer pathway linking primary metabolism to molecular oxygen (O_2_). They further positioned this pathway as an alternative respiratory route to the cytochrome chain [54,55]. With hindsight, the insight of these early researchers extended beyond the canonical cycle itself.

Szent-Györgyi’s 1928 use of H_2_S [52] as a quantitative DHA reductant is, to our knowledge, the earliest documentation of sulfide–DHA chemistry in the biochemical literature. It predates the work of Levenson, Rosen, and Hitchings (1951) [43] by more than two decades and the modern RSS framework by nearly a century [50,56]. In this sense, the three lineages traced below diverged from a single conceptual root that was already broader than the canonical ascorbate–glutathione cycle it eventually helped define. The question of whether non-thiol sulfur species participate directly in DHA reduction—raised implicitly by Szent-Györgyi’s 1928 observations [52] and largely set aside for the subsequent six decades—is precisely the question that the remainder of this review revisits.

### 2.2. The Plant Lineage

#### 2.2.1. Integration of ROS into the Foyer–Halliwell–Asada Pathway

Although Szent-Györgyi had already recognized in 1928 the chemical reactivity of ascorbate-related reducing systems toward peroxides [52], the biological relevance of ascorbate as an antioxidant was not fully appreciated until the discovery of superoxide dismutase (SOD) in 1969 [57]. The modern phase of plant ascorbate-recycling research began only after this conceptual gap had been filled by advances in the study of photooxidative stress [44,58]. Recognition of the highly reducing environment required for photosynthetic enzymes [59], together with growing interest in chloroplast pseudocyclic electron transport [60], shifted attention toward redox homeostasis in illuminated chloroplasts.

Within this context, Christine Foyer and Barry Halliwell reported in 1976 the presence of glutathione and glutathione reductase (GR) in chloroplasts and proposed a chloroplast pathway for the removal of superoxide (O_2_^•−^) and H_2_O_2_ [61]. This study is now recognized as a cornerstone of research on ascorbate-recycling mechanisms [47,48]. Kozi Asada and coworkers [62] subsequently made the catalytic requirement for DHA reduction explicit by identifying ascorbate-specific peroxidase (APX) in chloroplasts [63] and by characterizing DHAR as a thiol enzyme that can be stabilized in the presence of 2-mercaptoethanol (2-ME) [64]. The coupled enzymatic system that removes H_2_O_2_ using ascorbate, glutathione, and NADPH is referred to as the ascorbate–glutathione cycle or the Foyer–Halliwell–Asada pathway [47]. Without this recycling system, the chloroplast ascorbate pool has been estimated to be exhausted by the APX reaction within approximately 240 s [65]. Asada further expanded this framework by describing the photosynthetic ROS–scavenging system as the water–water cycle [58,62], a sequential electron-transfer pathway that links H_2_O oxidation at photosystem II (PSII) to APX-mediated detoxification of H_2_O_2_, ultimately regenerating H_2_O.

#### 2.2.2. The “Phantom Enzyme” Debate over DHAR

This canonical narrative did not, however, go unchallenged. The reduction of DHA by GSH, especially under alkaline conditions, was already recognized as a well-established chemical reaction [66]. This point is particularly relevant to chloroplasts, because the stromal pH rises to approximately 7.5 under illumination as a result of chemiosmotic H^+^ transport [61,67]. Given this favorable chemical environment, together with the highly labile nature of DHAR activity [61,64,68,69], the extent to which DHA reduction required a dedicated DHAR enzyme became a subject of active debate. Trümper et al. identified a novel DHAR from spinach chloroplasts, homologous to Kunitz-type trypsin inhibitors, that could reduce DHA using GSH [69]. In mammals, proteins such as glutaredoxin, also known as thioltransferase, and protein disulfide isomerase (PDI) have also been shown to possess DHA-reducing activity [66]. Morell et al. (1997) argued that much of the “DHAR activity” detected in plant extracts reflected nonspecific reduction by thiol–disulfide oxidoreductases rather than the action of a dedicated enzyme—an early articulation of what would later be called the “phantom enzyme” debate [48,70,71].

Functional and physiological evidence nonetheless continued to support a genuine role for DHAR. Yamasaki et al. (1995) reported a naturally occurring leaf-goldening phenotype in a tropical fig under high-light conditions [72]. This phenotype was associated with undetectable foliar DHAR activity and reduced ascorbate content [73], whereas photosynthetic activity and the activities of SOD, APX, and GR in the ascorbate–glutathione cycle were comparable to those of the wild type [74]. Further studies demonstrated that the mutant was susceptible to high light because of impaired non-photochemical quenching (NPQ) mediated by the xanthophyll cycle [75]. These observations provided early physiological evidence that DHAR contributes to photoprotection by maintaining the ascorbate supply required for violaxanthin de-epoxidase activity and the dissipation of excess excitation energy.

#### 2.2.3. Revisiting the “Phantom Enzyme” Debate in the Genomic Era

In the 21st century, genome projects advanced the molecular characterization of DHARs in land plants and clarified that they occur as small gene families across plant lineages [76]. These enzymes show close structural homology to members of the glutathione *S*-transferase (GST) superfamily and possess a conserved cysteine nucleophile [77]. *Arabidopsis thaliana* contains three functional DHAR-encoding genes, *DHAR1*, *DHAR2*, and *DHAR3*, together with two additional DHAR-like genes [78]. By analyzing the *Arabidopsis* single knockout mutants *dhar1*, *dhar2*, and *dhar3*, Noshi et al. (2017) showed that cytosolic DHAR1 is a major regulator of ascorbate and glutathione redox states under photooxidative stress [78].

In contrast, Rahantaniaina et al. (2017), using a complete set of single, double, and triple loss-of-function mutants for the three *Arabidopsis* DHARs, found no evidence for a major role of DHARs in maintaining ascorbate pools in plants grown under moderate light, either with or without intracellular oxidative stress [79]. Based on these findings, they suggested that these enzymes might be more appropriately regarded as glutathione dehydrogenases rather than as canonical DHARs [79]. In this interpretation, the enzymes may primarily catalyze GSH-dependent oxidation reactions rather than serving simply as dedicated DHA reductases. To account for the discrepancy between genetic and biochemical observations, they discussed the possible contribution of alternative DHA-reducing routes, including nonenzymatic mechanisms [79], and questioned whether DHA is indeed the major physiological oxidant of ascorbate in vivo [80]. In this sense, the “phantom enzyme” debate re-emerged in a modern genetic context.

By generating a quadruple mutant line combining the triple DHAR mutant with an additional *vtc2* mutation that causes ascorbate deficiency, Terai et al. (2020) demonstrated that DHARs make a non-negligible contribution to full ascorbate accumulation under high-light conditions, although they are dispensable when ascorbate concentrations are low to moderate [81]. They further examined the contribution of GSH-dependent chemical reduction using the *pad2* mutant, which contains reduced levels of GSH, and confirmed that DHAR function becomes important when light stress and ascorbate demand exceed a certain physiological threshold [81]. These findings are consistent with earlier physiological observations indicating that DHAR becomes particularly relevant when the demand for ascorbate recycling increases under excess light stress [73,74].

### 2.3. The Animal Lineage

#### 2.3.1. From Vitamin C to Candidate Recycling Enzymes

The animal lineage developed against a conceptual background fundamentally different from that of the plant lineage. Throughout much of the twentieth century, ascorbate in animal systems was studied primarily as “vitamin C”: a dietary requirement whose deficiency causes scurvy and whose isolation was achieved in the early 1930s [82]. This nutritional framing directed research attention mainly toward dietary intake, plasma and tissue distribution, and whole-organism turnover, as well as toward the antiscorbutic function of ascorbate as a cofactor for collagen prolyl and lysyl hydroxylases and other Fe- and Cu-dependent hydroxylases and monooxygenases [83]. In contrast to plant studies, in which antioxidant functions became a central focus, enzymatic recycling of ascorbate in animal systems developed within a different conceptual framework, shaped by the absence of the high-flux, illumination-driven ROS source that made recycling conspicuous in plants.

**Figure 1:**
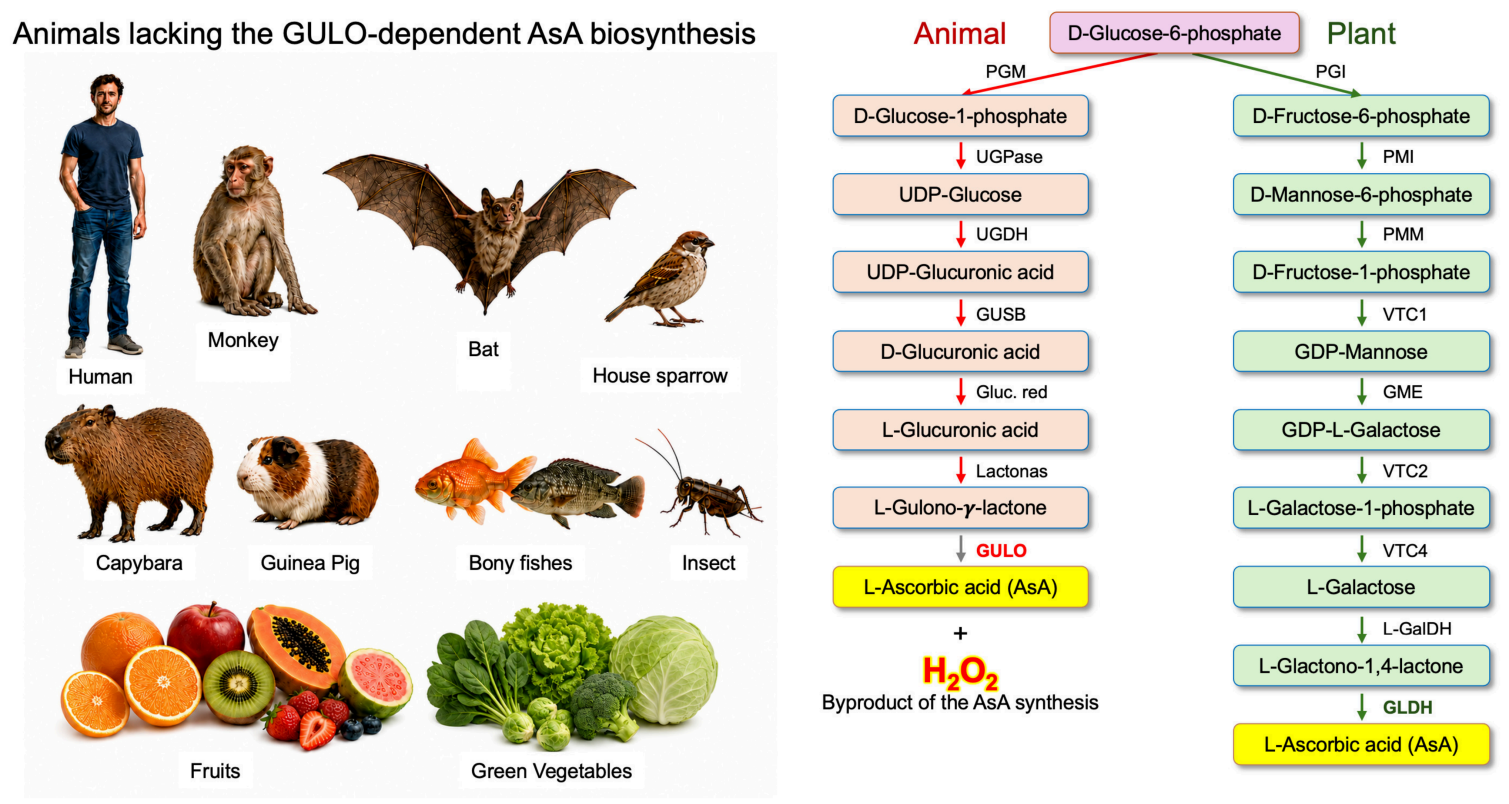
Loss of GULO across animal lineages and the divergent biosynthetic pathways of L-ascorbate in animals and plants. (**Left**) Representative animal lineages that have lost the ability to synthesize L-ascorbate (AsA) de novo due to loss-of-function of L-gulono-γ-lactone oxidase (GULO) [2,3,7]. These include humans and other haplorrhine primates (Old World monkey), capybara and guinea pig (Caviidae rodents), bats (Chiroptera), passerine birds (house sparrow), teleost fishes (goldfish, tilapia), and insects. All such species depend on exogenous supply from the diet to maintain their tissue AsA pools. Conventional dietary AsA sources are illustrated by fresh fruits and green vegetables. (**Right**) Comparison of L-ascorbate biosynthesis pathways in animals and plants [3,8]. Both pathways diverge from the shared metabolite D-glucose-6-phosphate. The animal uronate pathway (**left column**) proceeds through UDP-glucose, UDP-glucuronate, D-glucuronate, L-gulonate, and L-gulono-γ-lactone to AsA via GULO, a FAD-dependent oxidase localized to the smooth endoplasmic reticulum that generates H_2_O_2_ as a byproduct [1,3]. The plant Smirnoff–Wheeler pathway (**right column**) proceeds through D-fructose-6-phosphate, mannose phosphates, GDP-D-mannose, GDP-L-galactose, L-galactose-1-phosphate, L-galactose, and L-galactono-1,4-lactone to AsA via L-galactono-1,4-lactone dehydrogenase (GLDH), a cytochrome c-linked dehydrogenase of the mitochondrial inner membrane that does not produce H_2_O_2_ [9,10]. The GULO step in animals and the GLDH step in plants are both irreversible terminal commitments to AsA, and the GULO step is the locus of repeated evolutionary loss in several animal lineages incapable of de novo synthesis [2].

The question of how oxidized ascorbate is regenerated to its functional reduced form was framed differently, and pursued through a different set of experimental questions, for at least three reasons. First, most mammals synthesize ascorbate de novo from glucose, and the loss of L-gulono-γ-lactone oxidase (GULO) in humans, other primates, guinea pigs, and a few additional lineages (Figure 1) was historically regarded more as a dietary handicap than as evidence of a particular need for ascorbate-conservation machinery [2,7]. Second, animal cells lack the principal high-flux H_2_O_2_-generating context found in plants, namely photosynthetic electron transport [31]. The demand for rapid ascorbate regeneration was therefore less apparent at the whole-cell level, with activated neutrophils being a conspicuous exception [28]. Third, the development of clinical chemistry favored assays that measured “total ascorbate” after reducing DHA to ascorbate with chemical reagents in vitro [84,85]. Such assays were analytically useful, but they also sidestepped the question of how the same reduction is achieved in vivo.

#### 2.3.2. Candidate DHA-Reducing Systems in Animal Cells

The molecular phase emerged on a different timeline, shaped by the distinct experimental questions that framed animal ascorbate biology. Bigley and Stankova (1974) provided early evidence that mammalian leukocytes take up DHA and reduce it intracellularly [86]. Bigley et al. (1983) subsequently showed that this uptake is competitively linked to glucose transport in human neutrophils and fibroblasts, providing early evidence that DHA enters cells through glucose transport systems [87]. Ingermann et al. (1986) extended this observation to placental membrane vesicles, implicating a glucose-sensitive monosaccharide transporter [88], anticipating subsequent studies that connected DHA uptake with glucose-sensitive transport and ultimately led to the identification of facilitative hexose transporters as DHA carriers [89]. Washko and colleagues subsequently documented the capacity of human neutrophils to accumulate intracellular ascorbate to millimolar concentrations through this route [29,90]. Vera et al. (1993) then demonstrated, using heterologous expression systems, that mammalian facilitative hexose transporters mediate DHA uptake, thereby allowing intracellular accumulation of reduced ascorbate [89].

The first concrete mammalian DHA-reducing enzyme was reported by Park and Levine (1996), who purified DHA-reducing activity from human neutrophils and identified it as glutaredoxin (Grx), also known as thioltransferase [91,92]. A second NADPH-dependent route was described by May et al. (1997), who showed that thioredoxin reductase (TrxR) can also reduce DHA [93]. Earlier in vitro studies had also implicated PDI and hepatic 3α-hydroxysteroid dehydrogenase (3α-HSD) as candidate DHA reductases [66,94].

#### 2.3.3. Physiological DHA Reductases in Animal Cells: An Open Question

Despite these advances, no single dedicated enzyme directly analogous to plant DHAR has yet been firmly established in mammalian cells. Grx [91], TrxR [93], PDI [66], and 3α-HSD [94] have all been shown to possess DHA-reducing activity in vitro. However, each of these proteins was originally identified for a different primary function. Which of them, if any, constitutes the principal physiological DHA reductant in vivo—and in which tissues and under which conditions—therefore remains an open question.

### 2.4. The Chemistry Lineage

#### 2.4.1. Small-Molecule Reductants as Analytical Tools

The third lineage, nonenzymatic chemistry, is in some respects the oldest of the three. However, it developed from a fundamentally different perspective: small-molecule reductants of DHA were investigated primarily as analytical tools for quantifying ascorbate, rather than as candidate physiological reductants. By the mid-twentieth century, it was already well established that DHA could be reduced to ascorbate by a range of low-molecular-weight reductants. The reaction between GSH and DHA had been described qualitatively by Hopkins and Morgan (1936) and became central to the early formulation of ascorbate–glutathione coupling [53]. Cysteine (Cys) and other low-molecular-weight thiols were known to reduce DHA at appreciable rates [95]. As introduced in Section 2.1, Levenson, Rosen, and Hitchings (1951) had already demonstrated that H_2_S reduces DHA in aqueous solution [43]; viewed here from the chemistry lineage rather than the historical narrative, this finding can now be read in a new light following the modern recognition of H_2_S as a biological signaling molecule [96,97].

Subsequently, analytical studies came to rely on reducing reagents such as dithiothreitol (DTT) [98,99] and, later, tris(2-carboxyethyl)phosphine (TCEP) [84,100] to quantitatively reduce DHA before colorimetric or HPLC-based assays of “total ascorbate.” Homocysteine (Hcy) was likewise shown to reduce DHA chemically, although it has been used less commonly in routine biological assays [101,102].

#### 2.4.2. A 60-Year Dormancy

In these contexts, small-molecule reductants of DHA were understood primarily as in vitro reagents used to convert DHA to ascorbate before detection, rather than as candidates for the principal reductants operating in living cells. Levenson’s 1951 observation that H_2_S reduces DHA [43] was published in a clinical-chemical context that was largely disconnected from both plant photosynthesis and animal leukocyte biology. As a result, it appears to have remained outside the concerns of both fields for several decades—not through oversight, but because it had been framed as an analytical method rather than a mechanism. The DHA–sulfide reaction later acquired explicit physiological relevance in the work of Hildebrandt (2011) [103], who showed that DHA attenuates H_2_S toxicity and modulates sulfide oxidation in rat mitochondria. This finding reframed the chemical interaction between DHA and sulfide from an analytically useful reduction reaction to a potentially physiological mechanism.

### 2.5. Convergence on the RSS Question

These three lineages—canonical enzymatic systems in plants, candidate enzymatic systems in animals, and the solution chemistry of small-molecule reductants—advanced in near isolation for much of their respective histories. It should be noted, however, that the separation between the plant and animal lineages was not absolute. In plants, several noncanonical thiol-rich or redox-active proteins, including CPYC-type glutaredoxins, thioredoxin h (Trx h), defensins, metallothionein-like proteins, and Kunitz-type trypsin inhibitor-like proteins, have been reported to exhibit DHA-reducing activity in vitro [69,104,105,106]. Nevertheless, such activities do not necessarily establish these proteins as major physiological DHA reductases in vivo.

Plant studies have generally emphasized DHAR as a component of the ascorbate–glutathione cycle, whereas mammalian studies have emphasized Grx, TrxR, PDI, and related thiol–disulfide oxidoreductases as candidate DHA-reducing systems. Genetic analyses in *Arabidopsis* further suggest that non-DHAR thiol oxidoreductases are unlikely to account for the bulk of GSH-dependent DHAR activity under standard growth conditions, although their contributions may become relevant in specific tissues, developmental stages, or stress conditions [107,108].

Meanwhile, neither field paid sustained attention to the small-molecule chemistry of DHA reduction, which remained largely confined to analytical chemistry. The contemporary emergence of RSS biology [50,56], together with renewed quantitative interest in nonenzymatic DHA reduction [43,84,100,103], has now created an opportunity to place all three lineages in genuine dialogue (Figure 5). The central question raised by this convergence is therefore not simply whether DHA can be reduced, but what actually reduces DHA in vivo, under which cellular conditions, and to what extent RSS may constitute a missing component of ascorbate recycling.

## 3. Why Sulfur? The Chemical Basis of Thiol-Mediated DHA Reduction

The historical convergence described above raises a more basic chemical question. If plant DHARs, mammalian Grx/TrxR-type systems, GSH, Cys, H_2_S, and analytical reducing reagents all point repeatedly toward sulfur chemistry, this pattern may not be accidental. The issue is not whether sulfur is the only chemical route to DHA reduction, but why sulfur-containing compounds and cysteine-dependent proteins have so often emerged as effective DHA reductants. This section therefore turns from history to chemistry and asks what properties of sulfur make it particularly suited to the reduction of oxidized ascorbate species.

### 3.1. A Recurrent Pattern: Established DHA Reductants Often Involve Sulfur

A simple pattern emerges when DHA reduction is viewed across biological systems and chemical assays: many of the best-established reductants involve sulfur. In the ascorbate–glutathione cycle, DHA is reduced back to ascorbate mainly by glutathione (GSH), a sulfur-containing tripeptide thiol [48,58,61]. Enzymes that accelerate this reaction, such as DHARs and related Grx- or Trx-type proteins, also depend on reactive cysteine residues for catalysis [66,68,78,109,110]. In chloroplasts, this reaction helps maintain the ascorbate pool required for APX-dependent removal of photochemically generated H_2_O_2_ in the water–water cycle [58].

The same tendency is seen in vitro. Common reductants used to convert DHA back to ascorbate, including DTT, 2-mercaptoethanol, cysteine, homocysteine, and GSH, are thiols or sulfur-containing compounds [66,101]. This same H_2_S–DHA reaction (Section 2.4) is relevant today because RSS, such as persulfides and polysulfides, are now recognized as biologically important sulfur species with distinctive nucleophilic and redox properties [111,112,113].

This sulfur-centered pattern should not be overstated as an absolute rule. Other reductants can reduce DHA under appropriate chemical conditions, and the physiological relevance of any given reaction depends on concentration, compartmentation, pH, redox state, and reaction kinetics. Nevertheless, the repeated appearance of sulfur-containing reductants across enzymatic, cellular, and analytical contexts suggests that sulfur chemistry is especially well suited for DHA reduction.

### 3.2. DHA Reduction as a Two-Electron Reaction

The key point is that DHA reduction is not simply a matter of “adding electrons.” In aqueous solution, DHA exists predominantly as hydrated bicyclic forms rather than as the simple open-chain tricarbonyl structure, and its electrophilic carbon centers can react with nucleophiles such as thiols [114,115,116]. Thiols, especially their deprotonated thiolate forms (RS^−^), are effective nucleophiles under biological conditions [117]. DHA can react with thiol groups in low-molecular-weight thiols, peptides, and proteins, and reduced glutathione and related thiol systems can reduce DHA back to ascorbate with concomitant formation of disulfides or mixed disulfides [66,109,116]. In simplified form, the overall reaction can be written as:

DHA + 2 RSH → AsA + RSSR

This equation captures the essential logic of thiol-mediated DHA reduction. Two reducing equivalents are transferred from thiols to DHA, and the oxidized sulfur product is a disulfide. This is biologically useful because the reaction can proceed without requiring free radical intermediates. In cells, the resulting disulfides can then be reduced again by NADPH-dependent systems such as glutathione reductase or thioredoxin reductase [8,9].

### 3.3. Why Sulfur Works Well in This Reaction

Several chemical properties make sulfur particularly useful for DHA reduction, but they can be summarized simply. First, sulfur-containing thiols (RSH), especially their thiolate forms (RS^−^), are more reactive nucleophiles than the corresponding oxygen-based groups under physiological conditions [117,118]. This means that cysteine, GSH, and related thiols can react with DHA more readily than alcohols (ROH) or ordinary hydroxyl groups (–OH) [116].

Second, sulfur can form stable S–S bonds. This is important because the oxidation of two thiols to one disulfide provides a favorable end point for the reaction. Oxygen does not provide an equivalent pathway under biological conditions. This contrast is important because peroxide O–O bonds are relatively weak and readily undergo homolytic cleavage, making many peroxides chemically labile and reactive [119,120]. In this sense, sulfur is not only a donor of reducing equivalents but also provides a chemically convenient oxidized product.

Third, thiol/disulfide redox couples have reduction potentials that are well matched to the DHA/ascorbate couple (Figure 6). GSH, thioredoxin, glutaredoxin, and related systems are therefore strong enough to reduce DHA, while still being recyclable by cellular NADPH-dependent reductases. This makes sulfur chemistry both effective and biologically controllable.

### 3.4. From H_2_S to Reactive Sulfur Species

Historically, hydrogen sulfide was reported as a nonenzymatic reductant of DHA, in an early study by Levenson, Rosen, and Hitchings (1951) [43]. A thermodynamic analysis, however, makes free sulfide an unlikely reductant in this reaction. At pH 7, where the p*K*_a1_ of H_2_S (about 6.8 to 7.0) places it close to equal proportions of H_2_S and HS^−^, the two-electron couple in which sulfide is oxidized to elemental sulfur has a midpoint potential of about +0.17 V [125]. This value already reflects the H_2_S/HS^−^ mixture present at this pH, and it lies positive of the DHA/ascorbate couple (E°′ ≈ +0.09 V). Electron transfer from free sulfide to DHA is therefore thermodynamically uphill, irrespective of whether the species considered is H_2_S or its conjugate base HS^−^. The greater nucleophilicity of HS^−^ relative to H_2_S does not alter this conclusion, because nucleophilic reactivity is a kinetic property and does not confer the thermodynamic capacity to reduce DHA. On energetic grounds, free sulfide is a weak two-electron reductant. Although a large molar excess of sulfide can still drive measurable DHA reduction by mass action—consistent with the classical analytical observations—its unfavorable midpoint potential makes free H_2_S an inefficient regenerating agent relative to thiols.

A more coherent interpretation is that the reducing activity attributed to H_2_S reflects RSS formed within the sulfide system rather than the parent molecule. Persulfides (RSSH/RSS^−^), such as glutathione persulfide (GSSH), and inorganic polysulfides (H_2_S_n_/HS_n_^−^) are markedly more acidic and more nucleophilic than thiols or H_2_S. The p*K*_a_ of GSSH is near 5.45, compared with about 8.94 for GSH, so the persulfide is essentially fully ionized at physiological pH, and the resulting anion is intrinsically more reactive owing to the α effect [126,127]. These properties also make persulfides and polysulfides stronger reductants than the corresponding thiols, particularly in one-electron chemistry [56], and such species are now recognized as abundant in cells and tissues [50]. We therefore propose that the classical reduction of DHA in sulfide-containing systems is most plausibly mediated by RSS, in particular persulfides and polysulfides generated in situ, rather than by H_2_S itself. In a sulfide-containing solution, partial oxidation of H_2_S—for example by trace O_2_ or by DHA itself—readily generates inorganic polysulfides (H_2_S_n_/HS_n_^−^), so that even a nominally pure sulfide system contains a dynamic pool of more reactive sulfane-sulfur species. Unlike the clean thiol reaction, the two-electron oxidation product of a persulfide is not a simple disulfide but a higher sulfane-sulfur species; the detailed electron stoichiometry of DHA reduction by RSS therefore remains to be established. If DHA reduction favors soft, polarizable sulfur nucleophiles, some RSS may, in principle, be more kinetically competent than GSH or cysteine in specific cellular microenvironments, although direct experimental evidence for RSS-dependent DHA reduction remains limited. These properties are consistent with the emerging “supersulfide” framework proposed by Akaike, Sawa, and coworkers, in which sulfur species containing catenated sulfur atoms are increasingly recognized as abundant endogenous redox-active molecules across diverse organisms [128,129]. Within this framework, the lower p*K*_a_ and higher nucleophilicity of persulfides [50,126] are consistent with the possibility that some RSS act as kinetically competent reductants toward the electrophilic carbons of DHA (Section 3.2). It should be noted, however, that this advantage is clearer for one-electron radical chemistry, in which stabilized perthiyl radicals (RSS^•^) terminate rather than propagate radical chains [130,131], than for two-electron reactions, where the rate advantage over thiols is modest and persulfides are far less abundant in bulk pools [129]. Because DHA reduction is a two-electron process, any RSS contribution would most plausibly be restricted to local microenvironments where persulfide concentrations or pH transiently favor the more nucleophilic species. This scenario awaits direct experimental testing.

At present, this idea should be presented as a chemical hypothesis rather than as an established mechanism. Unlike the GSH/GSSG and DHA/ascorbate couples, standardized redox parameters for many RSS couples are not firmly established. Thus, the argument for RSS involvement currently rests more on their nucleophilicity, chemical reactivity, and biological abundance than on a fully defined thermodynamic framework.

In summary, the repeated appearance of sulfur-containing compounds in DHA reduction is unlikely to be coincidental. Thiols (RSH) provide a simple and recyclable route for the two-electron reduction of DHA, ending in disulfide formation. This same logic points naturally to RSS as potentially efficient DHA reductants, although their physiological contribution remains to be tested directly.

## 4. Multilayered Organization of Ascorbate Recycling Systems

The sulfur-centered chemistry discussed above helps explain why thiols and related sulfur species repeatedly appear as effective reductants of oxidized ascorbate species. However, chemistry alone does not explain how ascorbate recycling operates in living cells. In vivo, the same basic reduction reactions must be accelerated, localized, coupled to electron supply, and coordinated with cellular metabolism [48,132]. Thus, the next question is not only what can reduce DHA, but how these reactions are organized into a functional biological system.

From this perspective, ascorbate regeneration is best viewed not as a single pathway, but as a multilayered network. Basic chemical reactions are accelerated by enzymes, organized within cellular compartments and membranes, and integrated into broader redox and nutritional systems. This layered view (Figure 7) helps explain why similar reduction chemistry appears in bacteria, plants, and animals, while also highlighting unresolved questions about which reactions dominate in particular cellular contexts.

### 4.1. Layer 1: The Nonenzymatic Chemical Floor

The deepest layer consists of reactions that occur without enzymes. These reactions provide the chemical baseline upon which biological systems build. First, MDHA can spontaneously be disproportionate to ascorbate and DHA, thereby recovering part of the oxidized ascorbate pool. Second, DHA can be reduced by glutathione according to the reaction:

DHA + 2 GSH → AsA + GSSG

This reaction is relatively slow at physiological pH but establishes a universal thiol-dependent route for DHA reduction. Third, sulfide-containing systems have long been reported to reduce DHA to ascorbate in early chemical studies [43]. In light of current sulfur chemistry, this reaction may reflect not only free H_2_S/HS^−^ but also reactive sulfur species generated in situ.

### 4.2. Layer 2: Enzymatic Acceleration

The second layer consists of enzymes that accelerate these basic reactions and connect them to cellular reductant pools such as NAD(P)H and GSH. MDHA reductases catalyze one-electron recovery of MDHA [31,47]. In plants, NAD(P)H-dependent MDHAR and ferredoxin-dependent reduction in chloroplasts rapidly regenerate ascorbate [31]. In animals, cytochrome *b*561 and related membrane electron-transfer systems support MDHA reduction across membranes, as shown most clearly in adrenal chromaffin vesicles, where cytochrome *b*561 transfers electrons from cytosolic ascorbate to intravesicular MDHA [133,134,135,136].

DHA reduction is mainly a two-electron, thiol-dependent process. In plants, GSH-dependent DHAR is a central component of the ascorbate–glutathione cycle. In animals and bacteria, similar DHA-reducing activity is carried out by thiol–disulfide oxidoreductases such as Grx, PDI, and TrxR. Thus, Layer 2 does not create new chemistry; it accelerates and regulates the sulfur- and thiol-based chemistry already present in Layer 1.

### 4.3. Layer 3: Spatial Organization

The third layer concerns where ascorbate recycling occurs. In plants, ascorbate consumption is closely linked to photosynthesis and ROS production in chloroplasts, mitochondria, peroxisomes, and the cytosol [3,48]. Accordingly, MDHAR and DHAR isoforms are distributed among these compartments, allowing local maintenance of reduced ascorbate pools [11,79,108,137].

In animals, spatial organization follows a different logic. Many vertebrate cells can import DHA through glucose transporters and then reduce it intracellularly to ascorbate, making cytosolic DHA-reducing systems physiologically important [89,138,139]. In addition, membrane-bound electron-transfer proteins, particularly cytochromes *b*561, help maintain ascorbate pools across organellar or vesicular membranes by supporting transmembrane electron transfer coupled to MDHA reduction [136]. Recent findings that some ascorbate-related proteins can also function as redox-sensitive ion channels suggest that spatial organization is not static but can change in response to oxidative stress [140,141,142].

The AsA/DHA couple is not necessarily restricted to intracellular compartments. The caterpillar midgut lumen provides an unusual example of an extracellular AsA–GSH recycling system that buffers diet-derived oxidative pressure before peroxides enter epithelial cells [143]. Barbehenn et al. compared two moth caterpillars: the tannin-sensitive forest tent caterpillar, *Malacosoma disstria*, and the tannin-tolerant, white-marked tussock moth caterpillar, *Orgyia leucostigma*. In the tannin-sensitive caterpillar, ingested tannic acid is oxidized in the alkaline midgut lumen, producing high levels of peroxides, including H_2_O_2_. In the tannin-tolerant caterpillar, this oxidation is suppressed by higher luminal concentrations of ascorbate and glutathione [143]. This example extends ascorbate recycling beyond intracellular redox homeostasis to a boundary compartment, where the AsA–GSH couple acts as a first-line buffer against diet-derived prooxidants [35].

### 4.4. Layer 4: Network Integration and the RSS Frontier

The outermost layer integrates ascorbate recycling into broader cellular and dietary redox networks. In plants, the AsA–GSH cycle links ascorbate regeneration to GSH, NADPH, photosynthetic electron transport, and the oxidative pentose phosphate pathway [11,48]. However, NADPH supply is not restricted to this route alone: other NADPH-generating enzymes, most notably NADP-dependent isocitrate dehydrogenase (NADP-ICDH), also contribute to the cellular NADPH pool that supports antioxidant defense under stress conditions [144]. In animals, DHA reduction connects ascorbate recycling to GSH-, thioredoxin-, and NADPH-dependent redox systems [66,93].

Network integration is not restricted to sulfur- and NADPH-linked pathways. Ascorbate also participates in nonenzymatic reactions with a range of nitrogen oxidants: under acidic conditions, ascorbate reduces nitrite (NO_2_^−^) to nitric oxide (NO^•^), a reactive nitrogen species (RNS) that accumulates in acidified [41,42] or hypoxic microenvironments such as the stomach [6,145]. Ascorbate also reacts directly with *S*-nitrosothiols (RSNO), another class of RNS bearing a nitroso group on a thiol sulfur; depending on ascorbate concentration and the presence of trace copper ions, this reaction proceeds either through copper-catalyzed formation of NO^•^ and the corresponding disulfide (RSSR), or through direct nucleophilic attack at the nitroso-nitrogen, yielding free thiol, DHA, and NO^•^ [146]. Together, these nitrite- and RSNO-directed reactions parallel the nonenzymatic DHA-reducing chemistry discussed above (Section 3) and position ascorbate at the intersection of sulfur- and nitrogen-centered redox networks, consistent with the broader O_2_–NO^•^–H_2_S axis framework proposed elsewhere [44].

A major open question concerns the relationship between ascorbate recycling and RSS. The classical reaction between DHA and H_2_S shows that reduced sulfur can regenerate ascorbate chemically. More recently, persulfides and polysulfides have been recognized as abundant, reactive endogenous sulfur species with strong nucleophilic and reducing properties [50,51,126]. Because these molecules are more nucleophilic than ordinary thiols, they may be efficient DHA reductants. However, direct biochemical characterization of RSS-dependent DHA reduction remains limited or absent.

The endogenous status of these species is supported by dedicated biosynthetic routes: cysteinyl-tRNA synthetases generate cysteine persulfide and couple supersulfide production to mitochondrial bioenergetics [147], and this capacity appears evolutionarily conserved across bacteria, plants, and animals [148], consistent with the cross-kingdom perspective of this review. RSS are therefore not merely analytical reagents but constitutive components of cellular sulfur metabolism. Whether this endogenous pool contributes measurably to DHA reduction in vivo remains to be established experimentally.

This unresolved RSS frontier also raises a nutritional question. Plant-derived organosulfur compounds from *Allium* vegetables, such as garlic and onion, and Brassicaceae vegetables, such as broccoli and cabbage, can generate H_2_S- or polysulfide-related species in animal cells [149,150,151,152,153,154]. It is therefore possible, although still speculative, that dietary organosulfur compounds may influence vitamin C retention by supporting intracellular DHA reduction. In this view, ascorbate recycling extends from basic solution chemistry to enzymatic catalysis, cellular organization, redox-network integration, and potentially diet-derived sulfur metabolism (Figure 4).

## 5. The Ascorbate/DHA Couple as a Molecular Redox Capacitor

The multilayered organization described above shows that ascorbate recycling is not a single reaction, but a system that links chemistry, enzymes, compartments, membranes, and metabolic networks. The next step is to ask what this system accomplishes at the cellular level. One useful way to view the AsA/DHA couple is as a temporary reservoir of reducing capacity that absorbs fluctuations in oxidative demand and is then restored by recycling reactions. This idea can be illustrated by an analogy to an electrical capacitor.

### 5.1. A Circuit Analogy Already Familiar in Bioenergetics

Electrical analogies are not foreign to biology. Peter Mitchell’s chemiosmotic theory explains how ATP synthesis in respiration and photosynthesis is coupled to ion movement across energy-transducing membranes [155,156,157]. In this framework, energy transduction is described in terms of proton circuits, proton conductance, and the electrochemical proton gradient across membranes, expressed as Δμ_H_^+^ or the proton motive force (pmf) [67,157]. In that framework, proton (H^+^) movement is not treated merely as chemistry, but as a flow of stored and released energy. A similar systems-level view can also be applied to redox biology: electrons, like protons, are supplied, stored, transferred, and dissipated within organized cellular networks.

Here, we use the term redox capacitor as a simple analogy for the AsA/DHA recycling system. The analogy is conceptual, not literal. In an electrical circuit, a capacitor temporarily stores charge and releases it when demand suddenly increases or when excess current must be buffered (Figure 8). In cells, reduced ascorbate acts as a temporary reservoir of reducing equivalents. When ROS production suddenly increases, ascorbate donates electrons and is oxidized to MDHA and DHA. Recycling reactions then reduce MDHA/DHA back to ascorbate, restoring the reservoir.

### 5.2. Ascorbate Recycling as a Biological Redox Capacitor

Figure 9 applies this topology to intracellular redox biology. In the electrical circuit, the power supply provides electrons, the capacitor buffers current fluctuation, and the electronic device is protected. In the cellular redox circuit, NAD(P)H-dependent metabolism provides reducing power, ROS bursts represent transient oxidative overload, and macromolecules such as proteins, lipids, and nucleic acids are the protected targets [158].

The AsA/DHA recycling system is positioned between electron supply and oxidative demand. During ROS bursts, ascorbate is “discharged” by donating electrons to antioxidant reactions, including ascorbate peroxidase-dependent H_2_O_2_ reduction, direct ROS scavenging, and ascorbate-dependent reactions. The system is then “recharged” when MDHA and DHA are reduced back to ascorbate through MDHAR, DHAR, glutaredoxin-, thioredoxin-, GSH-, and NADPH-linked pathways, depending on the organism and compartment.

Thus, the AsA/DHA cycle is not simply a consumable antioxidant system. It is a rechargeable redox buffer that stabilizes intracellular electron flow under fluctuating oxidative load (Figure 9). The capacitor-like role of the AsA/DHA couple is not necessarily restricted to intracellular compartments. The caterpillar midgut lumen provides a rare example in which an extracellular AsA–GSH recycling system appears to buffer diet-derived oxidative pressure before peroxides diffuse into epithelial cells [143].

### 5.3. What Determines Redox Capacitance

**Figure 2:**
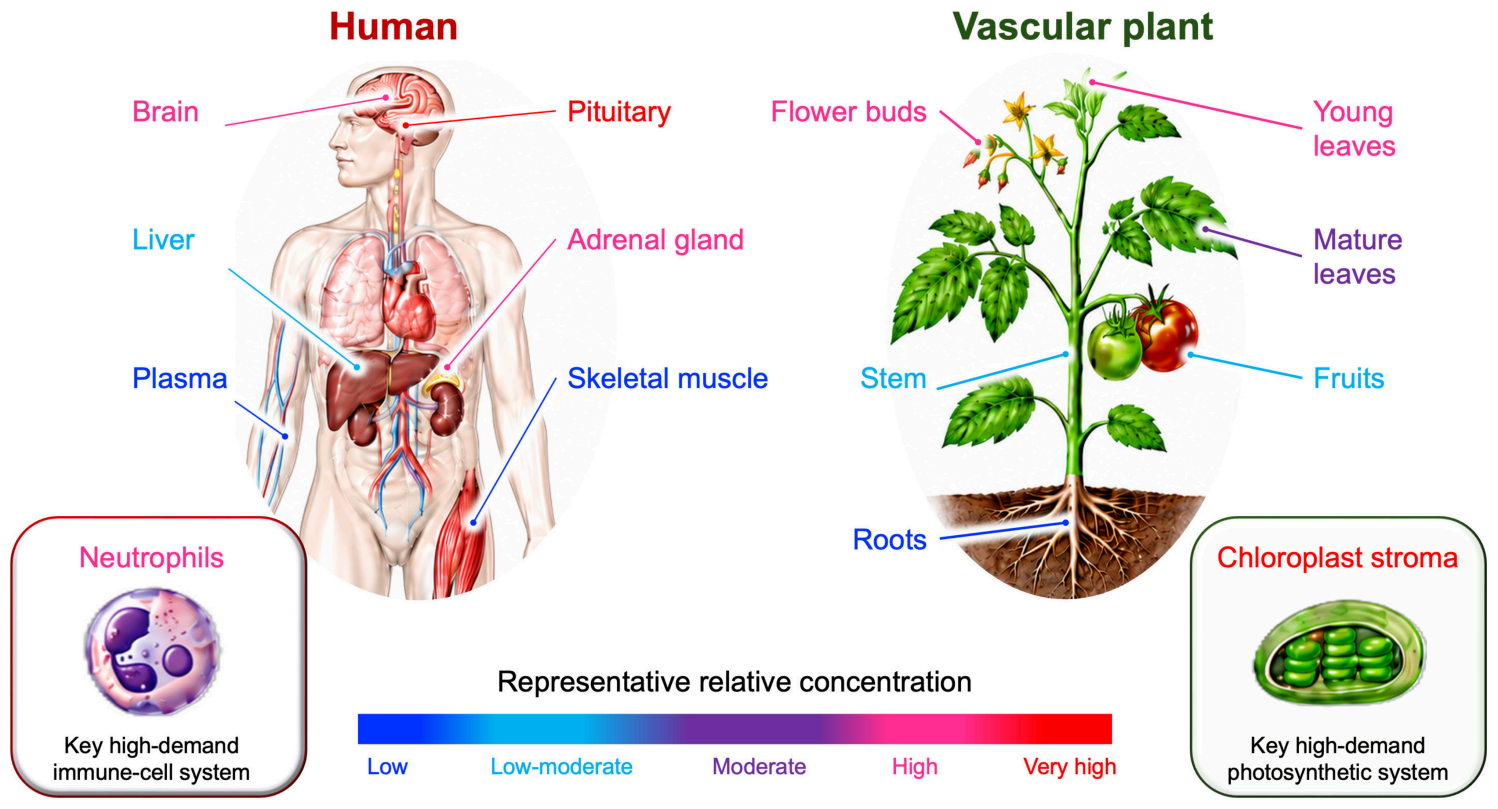
Relative distribution of ascorbate in human and vascular plant tissues. Ascorbate is unevenly distributed across organs, tissues, cell types, and subcellular compartments in both humans and vascular plants. Label colors indicate representative relative concentrations, ranging from low (blue) to very high (red). Insets highlight neutrophils and chloroplast stroma as high-demand immune-cell and photosynthetic compartments, respectively.

In this analogy, the redox capacitance of the AsA/DHA system depends mainly on three factors. First, it depends on the size of the reduced ascorbate pool (Figure 2). Chloroplasts contain high millimolar levels of ascorbate, consistent with their need to buffer sustained photooxidative pressure [31,58,159]. Neutrophils contain lower but still substantial ascorbate levels, suitable for short and intense respiratory bursts [29].

Second, it depends on the speed of recharging. A large ascorbate pool will be depleted if MDHA and DHA are not efficiently reduced back to ascorbate [65]. Therefore, MDHAR, DHAR, glutaredoxin, thioredoxin-related enzymes, GSH, and NADPH collectively determine the effective buffering capacity.

Third, it depends on how rapidly MDHA is recovered before DHA accumulates or undergoes further degradation. Efficient MDHA reduction helps preserve the reduced ascorbate pool and prevents irreversible loss from the cycle [58].

### 5.4. Why Ascorbate Is Suited to This Role

Ascorbate is well suited to act as a redox capacitor for three reasons. Its redox potential (Figure 3) allows it to reduce many biological oxidants without being rapidly wasted by direct reaction with molecular oxygen (O_2_). Its one-electron oxidation product, MDHA, is relatively stable, allowing enzymatic recovery rather than uncontrolled radical propagation. Finally, DHA can be enzymatically reduced back to ascorbate, making the system rechargeable.

This combination distinguishes ascorbate from other reductants. NADPH is the ultimate electron source, GSH is a major thiol redox buffer, and tocopherol protects membranes, but ascorbate uniquely combines water solubility, rapid oxidation, radical stability, and enzymatic regeneration.

### 5.5. A Shared Principle in Chloroplasts and Neutrophils

Chloroplasts and neutrophils experience very different oxidative challenges. Chloroplasts face sustained ROS formation during excess light [31], whereas neutrophils generate short but intense ROS bursts during host defense [28]. Nevertheless, both systems require a buffer placed between electron supply and oxidative overload (Figure 9).

The redox capacitor concept provides a common language for these two cases. It predicts that antioxidant protection depends not only on the total ascorbate concentration, but also on the rate of ROS production and the capacity for MDHA/DHA recycling. In this view, failure of ascorbate regeneration reduces cellular redox capacitance, even when ascorbate is initially abundant.

Although the specific term “molecular redox capacitor” has not, to our knowledge, been widely applied to the AsA/DHA couple, the concept builds on several established ideas: Mitchell’s proton-circuit analogy in bioenergetics [157], the description of ascorbate and glutathione as a cellular redox hub [11], quantitative concepts of redox buffer capacity [160], and biochemical analyses of the coupled AsA/DHA and GSH/GSSG systems [161].

## 6. Future Perspectives

### 6.1. DHAR, CLIC, and Moonlighting Redox Proteins

Returning to the capacitor framework introduced in Section 5, one of the most unexpected developments in ascorbate-recycling research (Figure 5) is the emerging connection between plant DHARs and chloride intracellular channel proteins (CLICs) as possible moonlighting redox proteins [48]. The term “moonlighting protein” was introduced by Constance Jeffery (1999) to describe multifunctional proteins in which a single polypeptide performs more than one physiologically relevant biochemical or biophysical function [162,163]. This concept was preceded by the idea of “gene sharing,” proposed for lens crystallins that also function as metabolic enzymes [164]. In current usage, moonlighting functions are distinguished from effects caused by gene fusion, alternative splicing, proteolytic fragments, isoforms, or nonspecific side activities [163,165].

Plant DHARs have traditionally been regarded as soluble glutathione-dependent enzymes that reduce DHA to ascorbate in the ascorbate–glutathione cycle. However, their membership in the glutathione *S*-transferase (GST) superfamily suggests potential functions beyond classical DHA reduction. A parallel case is provided by mammalian CLIC1, which was first cloned as a nuclear chloride channel protein, later shown to possess a soluble GST-like fold, and subsequently recognized as a redox-regulated dimorphic protein that can shift between soluble and membrane-associated states [140,166]. Under oxidative stress, CLIC1 translocates from the cytosol to the plasma membrane, where its anionic conductance has been proposed to balance the charge displaced by NADPH oxidase activity, illustrating how a single protein can act as both a redox sensor and a membrane effector [167]. Al Khamici and coworkers (2015) demonstrated that CLIC proteins possess glutaredoxin-like, GSH-dependent oxidoreductase activity, including DHA-reducing activity in the case of human CLIC1 [141]. Das and coworkers (2022) subsequently provided detailed kinetic analyses showing that human CLIC1, CLIC3, and CLIC4 catalyze GSH-dependent DHA reduction, with crystal structures capturing distinct oxidation states of the catalytic cysteine [168]. These findings suggest that the boundary between “redox enzyme” and “ion channel” may be less rigid than previously assumed.

A similar possibility has now entered plant biology [48]. *Arabidopsis* DHAR1 was reported to generate ion conductance in heterologous systems, and pearl millet DHAR was recently proposed to moonlight as a membrane-integrated ion channel [142,169]. These observations do not imply that all plant DHARs primarily function as channels in vivo, or that DHA reduction and ion conductance occur under the same conditions. Rather, they raise a testable hypothesis: under moderate oxidative load, DHAR may support the AsA/DHA redox couple as part of a cellular redox capacitor, whereas under stronger or more localized oxidative stress, conformational change, membrane association, or oligomerization may shift part of the protein population toward ion conductance. Such a transition would connect an electron-transfer circuit based on ascorbate recycling with an ion/proton circuit related to membrane potential, pH homeostasis, and chemiosmotic coupling [67].

This framework remains speculative and requires direct experimental testing. Key questions include whether native plant DHARs insert into specific membranes under physiological stress, whether this process is reversible, which cysteine residues control the transition, what ions are conducted, and whether ion flow affects chloroplast, mitochondrial, vacuolar, or plasma-membrane proton gradients. This question is relevant to the broader view that localized proton availability and proton transfer across membranes can regulate H_2_O_2_ (ROS)-, NO^•^ (RNS)-, and H_2_S (RSS)-related reactions and controlled uncoupling in biological systems [67]. If confirmed, DHAR/CLIC-like moonlighting would broaden the concept of ascorbate recycling from a soluble enzymatic reaction to a dynamic redox–membrane system capable of linking antioxidant recycling, ion transport, and possibly controlled dissipation of redox pressure under stress.

### 6.2. Could Dietary Sulfur Compounds Influence Ascorbate Regeneration

An open question emerging from this review is whether RSS contribute to ascorbate regeneration beyond the established glutathione-dependent pathways. As established in Section 2 and Section 3, DHA reacts with H_2_S to regenerate ascorbate [43], implying that more nucleophilic sulfur species, such as persulfides and polysulfides, deserve particular attention. These species are now recognized as endogenous redox-active molecules in both animals [170] and plants [171], but their possible contribution to DHA reduction has not yet been systematically examined.

This question is especially relevant because plants contain many sulfur-rich secondary metabolites that can serve, directly or indirectly, as sources of H_2_S, persulfides, or polysulfides. Examples include allicin and diallyl sulfides from *Allium* species, *S*-allyl cysteine from garlic preparations [149,151], and isothiocyanates such as sulforaphane from Brassicaceae [172]. These compounds originally function as chemical defenses against microbes and herbivores [152], yet many are also associated with health-promoting effects in animals and humans [151]. Their mechanisms are usually discussed in terms of electrophilic signaling, Nrf2 activation, detoxification enzymes, mitochondrial protection, or inflammatory control [173,174]. However, part of their biological activity may also reflect a simpler redox contribution: supplementation of sulfur-based reducing capacity that helps maintain the ascorbate pool, particularly under oxidative stress.

From an evolutionary perspective, this possibility raises a broader question. Animals that cannot synthesize ascorbate, including humans and other GULO-deficient lineages, are usually viewed as dependent on continuous dietary vitamin C intake. Yet animals may also ingest sulfur compounds that support ascorbate recycling rather than simply supply ascorbate itself. In this sense, the nutritional value of plant foods may not be limited to their vitamin C content; it may also include their capacity to sustain ascorbate regeneration through RSS chemistry. Testing this idea will require comparative studies across GULO-retaining and GULO-deficient animals, with attention to diet, sulfur metabolism, tissue DHA-reducing activity, and the enzymes controlling RSS turnover, such as CBS, CSE, 3-MST, SQR, and ETHE1 [97,175]. Such studies could clarify whether sulfur-rich diets merely accompany ascorbate biology, or whether they partly compensate for the loss or limitation of endogenous ascorbate synthesis.

### 6.3. Gut Microbial H_2_S and Local Ascorbate Redox Balance: A Testable Hypothesis

A second possible source of sulfur-based reductants is the gut microbiota. In the colon, sulfate- and sulfite-reducing bacteria (SRB), as well as cysteine-degrading commensals, produce H_2_S from inorganic sulfate, taurine, cysteine, methionine, and other sulfur-containing substrates [176,177]. This microbial H_2_S is usually discussed in relation to mucosal toxicity, epithelial metabolism, inflammation, and host defense [177]. However, from the perspective of ascorbate recycling, a more limited but testable possibility can be proposed: microbial H_2_S, and possibly related RSS, may contribute to local extracellular reduction of DHA to AsA in the intestinal lumen or at the mucosal surface.

**Figure 4:**
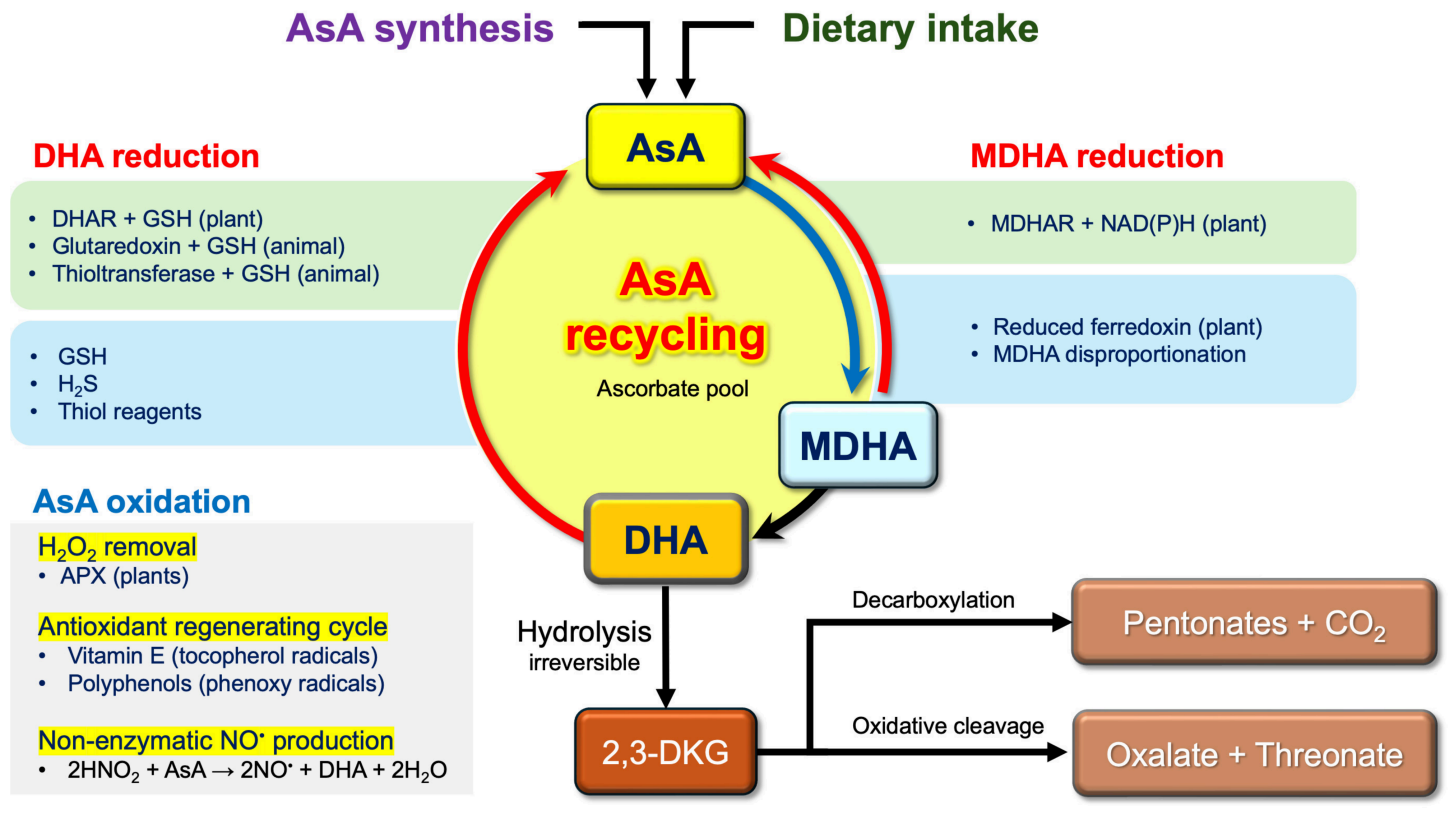
Overview of ascorbate recycling and degradation pathways. Ascorbate (AsA) is supplied by de novo biosynthesis or dietary intake and functions as a central antioxidant pool. Oxidation of ascorbate generates monodehydroascorbate (MDHA) and dehydroascorbate (DHA), which can be recycled back to AsA through MDHA- and DHA-reducing systems. MDHA reduction can be mediated by MDHAR using NAD(P)H or reduced ferredoxin, or by spontaneous disproportionation, whereas DHA reduction involves enzymatic systems such as DHAR, glutaredoxin, and thioltransferase, as well as low-molecular-weight reductants including GSH, H_2_S, and thiol reagents. When DHA is not efficiently recycled, it undergoes irreversible hydrolysis to 2,3-diketogulonate (2,3-DKG), followed by further degradation to products such as pentonates, CO_2_, oxalate, and threonate. AsA oxidation can also be linked to H_2_O_2_ removal by APX [39], to regeneration of antioxidant molecules such as vitamin E [33] and polyphenols [40], and to nonenzymatic nitric oxide (NO^•^) production [41,42].

This possibility should be framed cautiously. DHA is chemically unstable in aqueous solution and can be irreversibly hydrolyzed to 2,3-diketogulonic acid (Figure 4); therefore, any contribution of microbial H_2_S to ascorbate regeneration would need to occur rapidly and locally, before DHA degradation. The most plausible site is not the bulk colonic lumen, but the mucosal microenvironment, where microbial metabolites, mucus components, epithelial transporters, host antioxidants, and immune-cell-derived oxidants coexist. In this setting, controlled H_2_S production might help maintain a local extracellular AsA/DHA balance, whereas excessive sulfidogenesis could instead damage epithelial cells, inhibit mitochondrial respiration, promote genotoxic stress, and aggravate inflammation [178]. Thus, the relevant question is not whether microbial H_2_S is simply beneficial or harmful, but whether a regulated range of sulfur metabolism contributes to mucosal redox buffering (Figure 10).

## 7. Conclusions

Ascorbate biosynthesis appears to have been established in photosynthetic eukaryotes after cyanobacterial plastid endosymbiosis, in parallel with oxygenic photosynthesis and the increasing burden of ROS [9,179]. In plants and algae, ascorbate is therefore closely linked to photosynthetic redox control. Its major functions include H_2_O_2_ detoxification by APX, violaxanthin de-epoxidation in non-photochemical quenching (NPQ), and operation of the ascorbate–glutathione cycle [3,11,58,108,180]. These reactions reflect an ancestral role of ascorbate as a high-capacity, regenerable antioxidant solute.

In animals, ascorbate acquired a different set of functions. It supports collagen hydroxylation and connective-tissue maturation [181]; carnitine biosynthesis through ascorbate-dependent trimethyllysine and γ-butyrobetaine hydroxylation reactions [182]; catecholamine biosynthesis through dopamine β-hydroxylase-dependent norepinephrine formation [183]; hypoxia-inducible factor-α (HIF-α) hydroxylation as part of cellular oxygen sensing [184,185]; and epigenetic regulation through ten-eleven translocation (TET)-mediated DNA demethylation and Jumonji C (JmjC)-dependent histone demethylation [186]. These reactions depend on Fe^2+^/2-oxoglutarate-dependent dioxygenases, in which ascorbate maintains the catalytic iron center in the reduced state [185,187].

Thus, plant and animal ascorbate biology share a common redox logic, but use it for different physiological outputs: photoprotection and redox buffering in plants, and connective tissue formation, metabolism, signaling, and gene regulation in animals.

This review has argued that vitamin C biology should not be framed only as a question of dietary intake. The familiar clinical story—that humans lost gulonolactone oxidase, cannot synthesize ascorbate, and therefore require vitamin C from food—is important, but it is only one part of a larger problem. In all organisms that use ascorbate, the key issue is how cells maintain a reduced and functional ascorbate pool despite continuous oxidation to MDHA and DHA.

Ascorbate recycling can be viewed as a redox circuit that prevents the loss of this pool. MDHAR, DHAR, glutaredoxin, thioltransferase systems, and glutathione are established contributors, whereas sulfide-containing systems and RSS remain chemically plausible but experimentally unresolved candidates (Figure 4). In this sense, the ascorbate pool functions like a cellular redox capacitor (Figure 9): it absorbs oxidative pressure, stores reducing capacity in a readily usable form, and releases it when local reactions require electrons (Figure 8). The biological value of ascorbate therefore depends not only on how much is synthesized or ingested, but also on how efficiently it is regenerated.

The multilayered model proposed in this review (Figure 7) provides a simple way to understand this system. At the chemical level, DHA can be reduced by thiols, sulfide-containing systems, and related reductants. At the enzymatic level, proteins such as DHAR and glutaredoxin accelerate and control these reactions. At the spatial level, these reactions are organized in chloroplasts, cytosol, mitochondria, apoplast, membranes, and extracellular or intestinal environments. At the network level, ascorbate recycling is connected to NAD(P)H, glutathione, sulfur metabolism, and possibly RSS signaling. These layers are not separate alternatives; they work together to stabilize the ascorbate pool under changing redox conditions.

**Figure 5:**
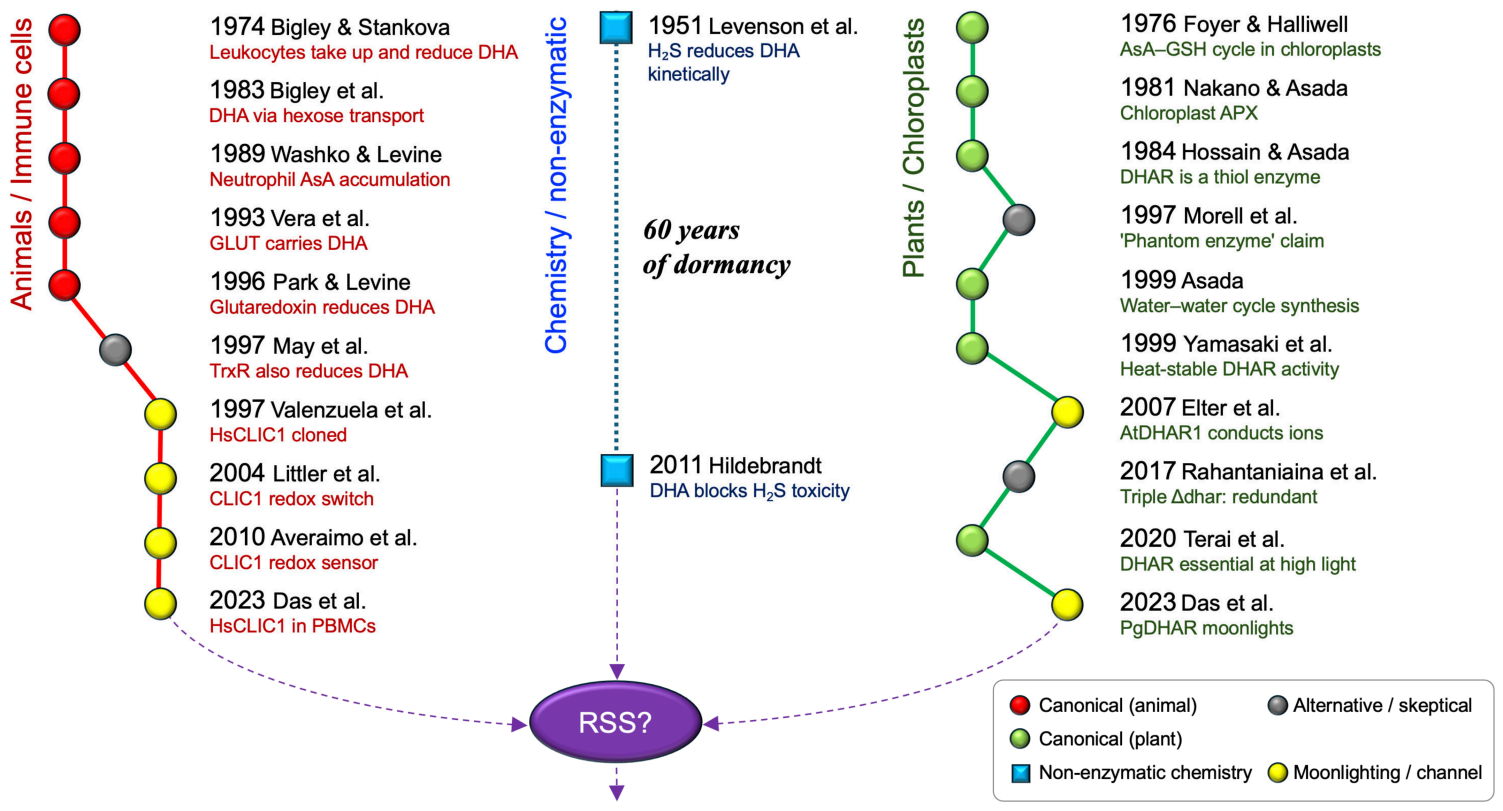
Three converging research traditions in dehydroascorbate (DHA) reduction. A chronological synthesis of key findings on ascorbate recycling, arranged as three parallel tracks. Animal, plant, and solution-chemistry studies developed largely independently but now converge on the unresolved question of how DHA is reduced in vivo.

**Figure 6:**
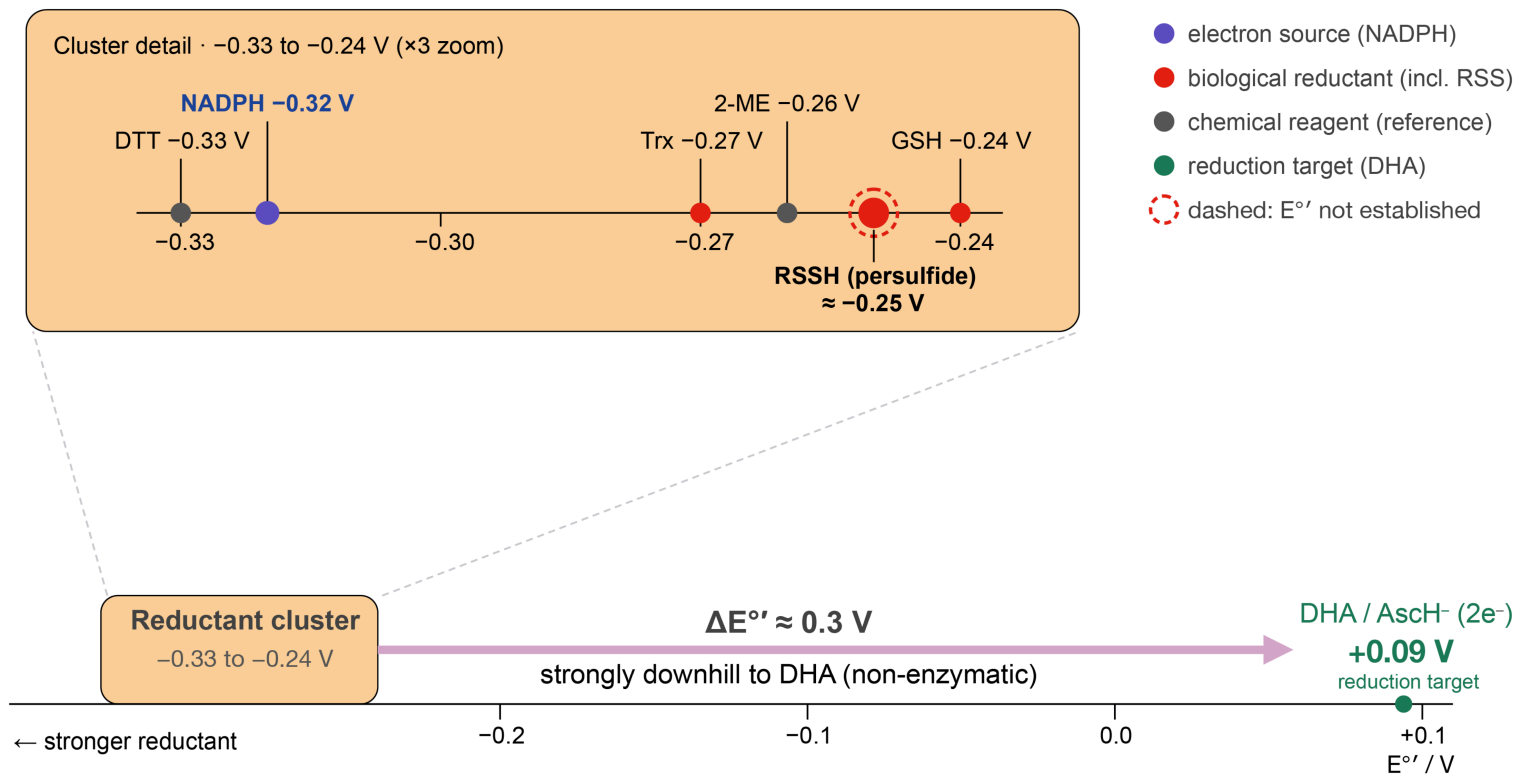
Reactive sulfur species lie within the reductant potential window suited to dehydroascorbate (DHA) reduction. Standard midpoint potentials (E°′, pH 7) are plotted on a single horizontal scale, with more negative values (stronger reductants) to the left and more positive values (stronger oxidants) to the right. The two-electron DHA/ascorbate couple (DHA/AscH^−^) sits at +0.09 V (green, **right**), whereas the biological and chemical reductants relevant to nonenzymatic DHA reduction cluster between −0.33 and −0.24 V (box, **left**). The horizontal separation between the two corresponds to a driving force of ΔE°′ ≈ 0.3 V, indicating that DHA reduction by these reductants is thermodynamically favorable and can, in principle, proceed nonenzymatically; the arrow marks this potential gap. The inset (×3 expansion of the −0.33 to −0.24 V window) resolves the individual couples: DTT-S_2_/DTT(SH)_2_ (−0.33 V) and (2-ME)_2_/2-ME (−0.26 V) as chemical reference reductants; NADP^+^/NADPH (−0.32 V) as the ultimate electron source; and the biological reductants Trx-S_2_/Trx(SH)_2_ (−0.27 V), GSSG/2 GSH (−0.24 V), and the persulfide couple RSS_n_/RSSH (≈−0.25 V). The persulfide (RSSH), the focus of this review, falls in the same narrow band as glutathione and thioredoxin, identifying it as thermodynamically competent for DHA reduction. Its potential is drawn with a dashed marker because a formal E°′ value has not been established; the position (≈−0.25 V) is therefore approximate and effectively iso-potential with the GSSG/2 GSH couple. Color coding: purple, electron source (NADPH); red, biological reductant (including RSS); gray, chemical reagent; green, reduction target (DHA); dashed outline, E°′ not established. Standard midpoint potentials (E°′, pH 7) are taken from the following sources: DTT-S_2_/DTT(SH)_2_, −0.33 V (Lamoureux and Whitesides, 1993 [121]); NADP^+^/NADPH, −0.32 V (Noctor, 2006 [32]); Trx-S_2_/Trx(SH)_2_, −0.27 V, value for Escherichia coli thioredoxin (Åslund et al., 1997 [122]); (2-ME)_2_/2-ME, −0.26 V (Lees and Whitesides, 1993 [123]); GSSG/2 GSH, −0.24 V (Schafer and Buettner, 2001 [124]); RSS_n_/RSSH, ≈ −0.25 V, an estimate because a formal E°′ has not been established (Filipovic et al., 2018 [56]); DHA/AscH^−^, +0.09 V, midpoint potential at pH 7 (Noctor, 2006 [32]). The two-electron DHA/AscH^−^ midpoint reported in the literature spans roughly +0.05 to +0.10 V. The value implied by the one-electron couples in Figure 3 (mean of +0.282 V and −0.174 V = +0.054 V) and the +0.09 V adopted here from Noctor (2006) [32] both fall within this range; +0.09 V is used consistently throughout the main text and Figure 6. The reduction potentials of the thiol and dithiol reagents are not measured directly but are derived from thiol-disulfide interchange equilibria (Lees and Whitesides, 1993 [123]).

**Figure 7:**
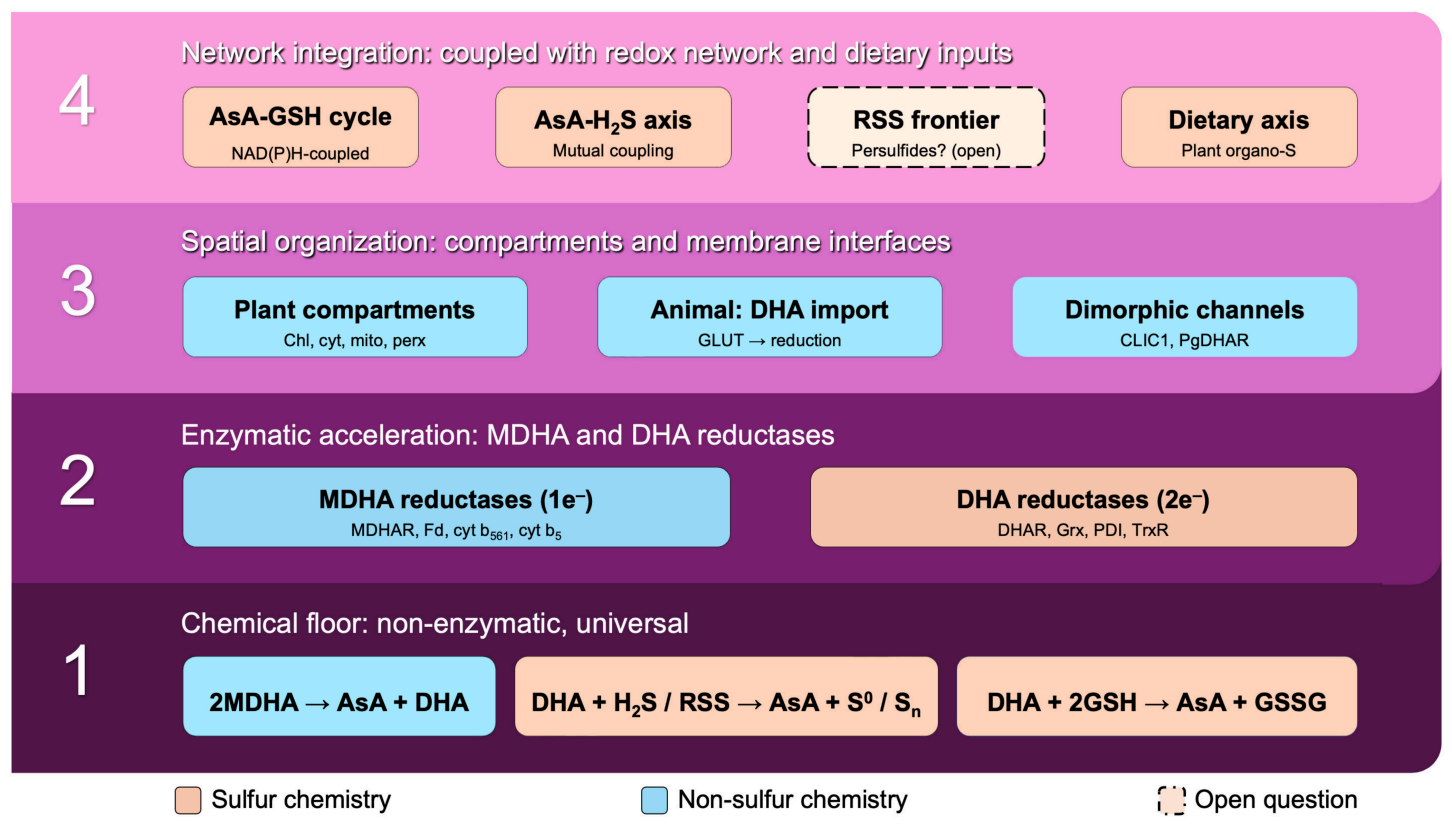
Multilayered organization of ascorbate recycling in biological systems. Ascorbate recycling can be viewed as a four-layered system. Layer 1 represents the nonenzymatic chemical floor, including spontaneous MDHA disproportionation and sulfur- or thiol-dependent DHA reduction. Layer 2 consists of enzymatic acceleration by MDHA reductases and DHA reductases. Layer 3 adds spatial organization through plant organelles, animal DHA import systems, and membrane-associated redox proteins or dimorphic channels. Layer 4 integrates AsA recycling with broader redox networks, the AsA–H_2_S axis, unresolved RSS chemistry, and possible dietary inputs from plant organosulfur compounds. In Layer 1, the sulfide-dependent reaction is shown with H_2_S/RSS to indicate that, as discussed in Section 3.4, the effective reductant is most plausibly RSS generated in situ rather than free H_2_S. Peach-colored boxes denote sulfur-based reactions or systems, blue boxes denote non-sulfur reactions or systems, and dashed outlines mark unresolved or hypothetical components.

**Figure 8:**
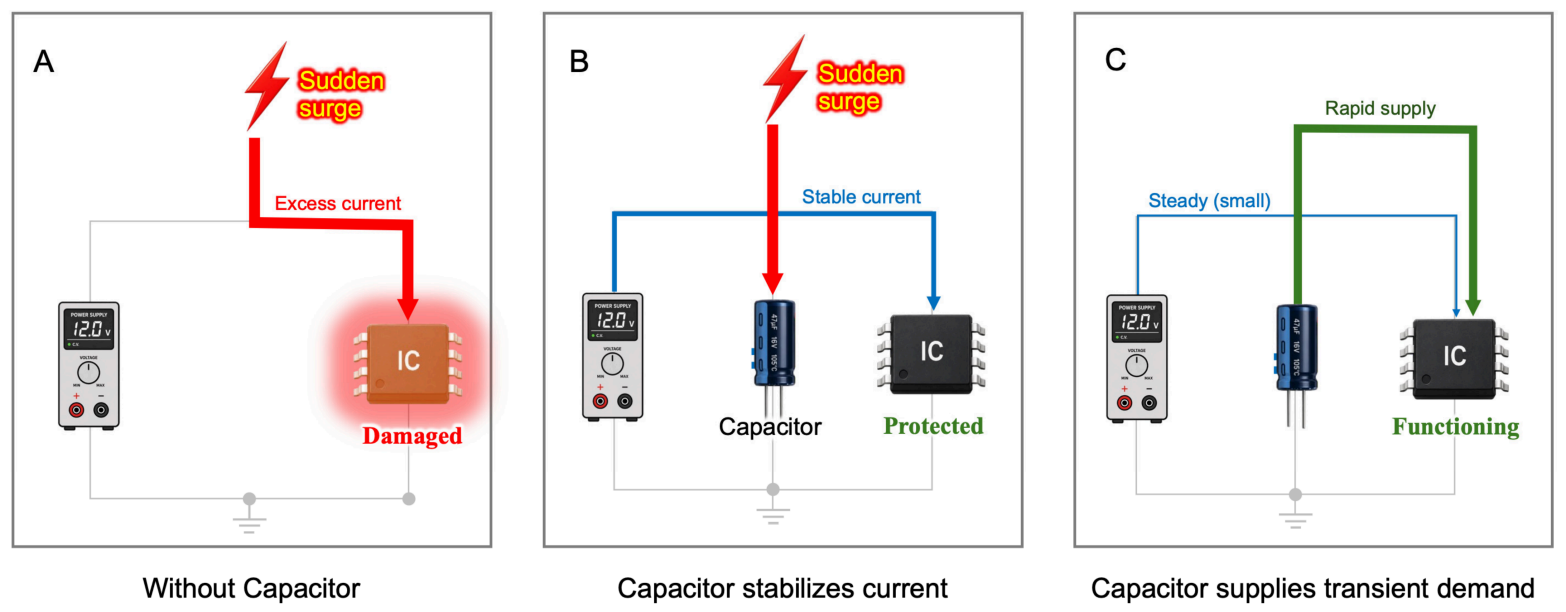
What a capacitor does in a simple circuit. A capacitor stabilizes a circuit by temporarily storing and releasing charge. Without a capacitor, a sudden surge can directly damage the target device (**A**). A capacitor can buffer excess current and protect the device (**B**), and it can also supply current rapidly when transient demand exceeds the steady power supply (**C**). This dual function—absorbing excess and supplying demand—is the key point of the analogy. The capacitor does not create energy; it temporarily stores and releases it to stabilize the whole system.

**Figure 9:**
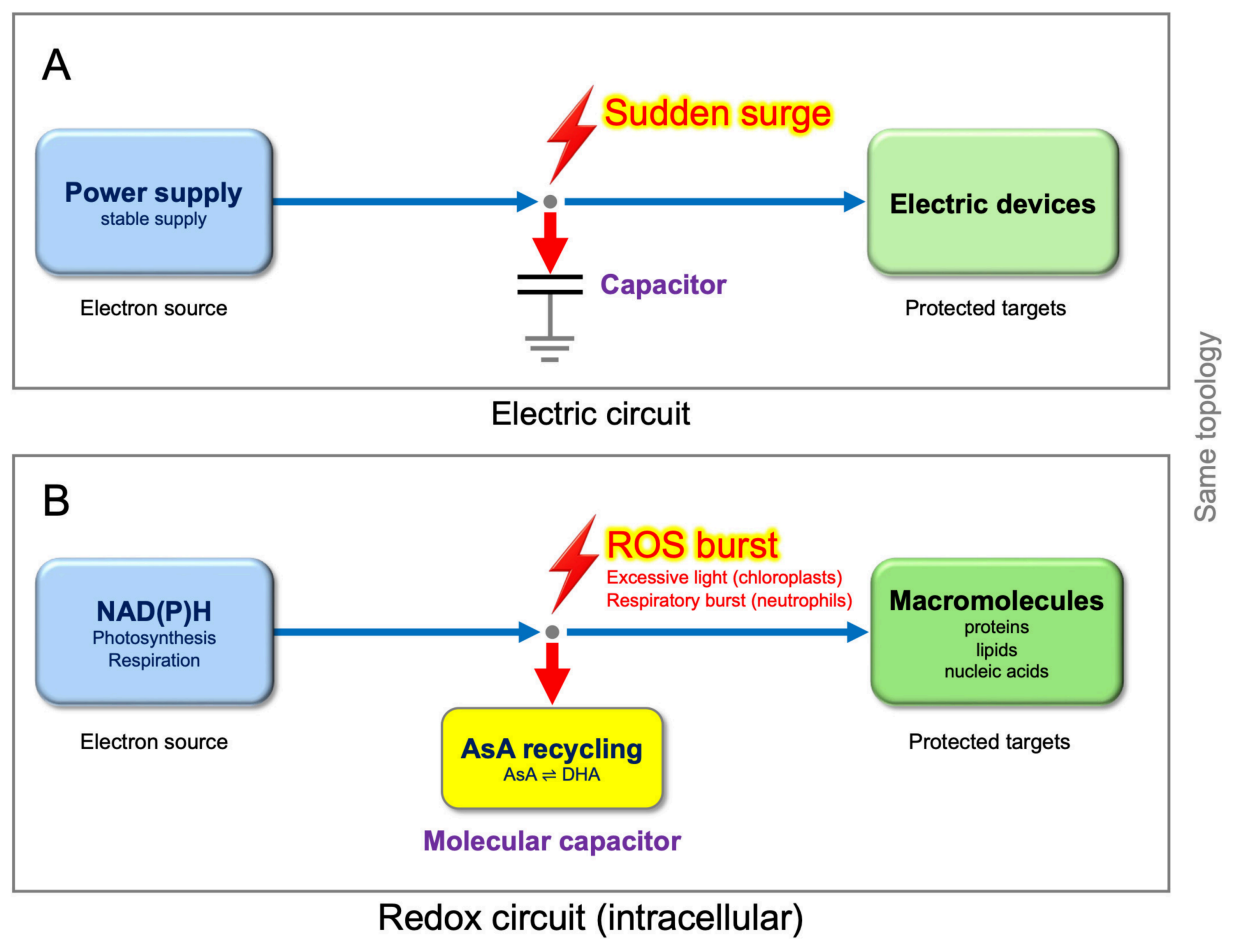
Ascorbate recycling as a biological redox capacitor. (**A**) Electrical circuit. A capacitor positioned between the power supply and electrical devices buffers a sudden surge and protects downstream components. (**B**) Intracellular redox circuit. The same topology can be applied to an intracellular redox circuit. NAD(P)H-dependent metabolism provides reducing power, ROS bursts represent transient oxidative overload, and macromolecules are protected targets. The AsA/DHA recycling system is positioned between electron supply and oxidative demand. Ascorbate is oxidized during ROS detoxification and then regenerated through MDHA/DHA-reducing systems, allowing the cycle to function as a rechargeable redox buffer.

**Figure 10:**
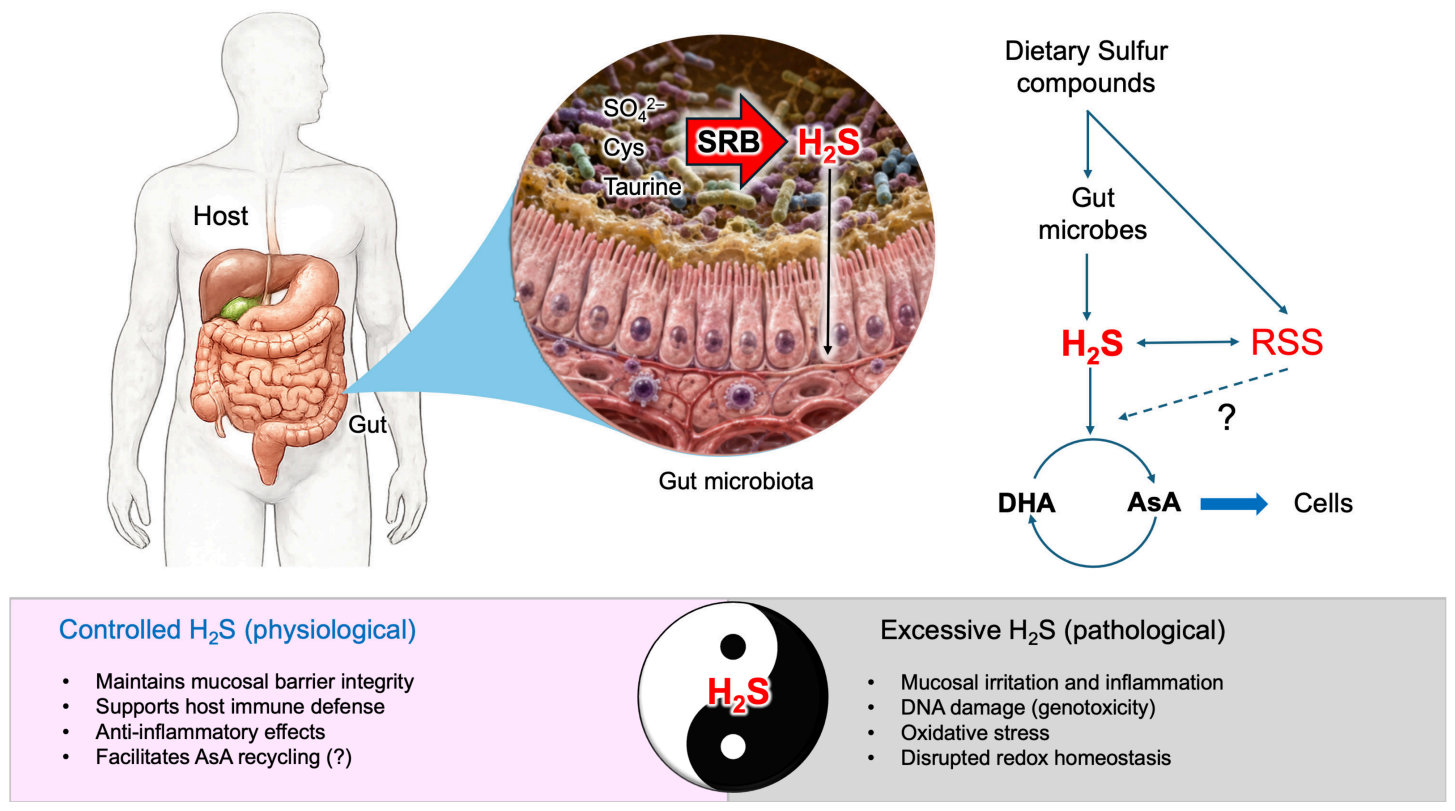
Gut microbial H_2_S as a possible local input to extracellular ascorbate recycling. Sulfate-reducing bacteria (SRB) and other gut microbes generate H_2_S from sulfate, cysteine, taurine, and dietary sulfur compounds. H_2_S and related reactive sulfur species (RSS), including persulfides, may locally reduce DHA to AsA in the intestinal lumen or at the mucosal surface, although this remains hypothetical. Physiological H_2_S may support mucosal barrier function, host defense, and redox buffering, whereas excessive H_2_S can cause inflammation, genotoxicity, oxidative stress, and redox imbalance.

This view also gives new meaning to the long and fragmented history of DHA reduction research (Figure 5). Early chemical studies [43], plant ascorbate–glutathione cycle research [47], animal thioltransferase and glutaredoxin studies [66], recent findings on DHAR/CLIC moonlighting behavior [142], and emerging interest in RSS [44,170,188,189] can all be seen as different windows into the same problem: how cells regenerate ascorbate fast enough to support oxygen-dependent life.

A broader nutritional perspective follows from this conclusion. Vitamin C status may depend not only on ascorbate intake [187], but also on dietary and metabolic factors that support ascorbate recycling. These may include RSS-related reactions. The intestinal microbiota may also influence this redox environment by modifying sulfur metabolism and producing or consuming redox-active compounds such as persulfides and polysulfides. Future vitamin C research should therefore move beyond the question of “how much vitamin C is consumed” and ask how diet, sulfur metabolism, host redox enzymes, and the microbiome together maintain the regenerated ascorbate pool.

## Data Availability

No new data were created or analyzed in this study. Data sharing is not applicable.

## Abbreviations

The following abbreviations are used in this manuscript:

2,3-DKG	2,3-Diketogulonate
2-ME	2-Mercaptoethanol
3α-HSD	3α-Hydroxysteroid dehydrogenase
3-MST	3-Mercaptopyruvate sulfurtransferase
APX	Ascorbate peroxidase
AsA	L-Ascorbic acid
CBS	Cystathionine β-synthase
CLIC	Chloride intracellular channel
CLIC1	Chloride intracellular channel 1
CSE	Cystathionine γ-lyase
DBH	Dopamine β-hydroxylase
DHA	Dehydroascorbic acid
DHAR	Dehydroascorbate reductase
DTT	Dithiothreitol
ETHE1	Ethylmalonic encephalopathy protein 1; also known as persulfide dioxygenase
GLDH	L-Galactono-1,4-lactone dehydrogenase
GME	GDP-mannose-3′,5′-epimerase
GR	Glutathione reductase
GSH	Reduced glutathione
GSSG	Oxidized glutathione; glutathione disulfide
GST	Glutathione *S*-transferase
GULO	L-Gulono-γ-lactone oxidase
GUSB	β-Glucuronidase
Grx	Glutaredoxin
HIF-α	Hypoxia-inducible factor-α
Hcy	Homocysteine
H_2_S	Hydrogen sulfide
H_2_O_2_	Hydrogen peroxide
JmjC	Jumonji C
L-GalDH	L-Galactose dehydrogenase
MDHA	Monodehydroascorbic acid
MDHAR	Monodehydroascorbate reductase
NO^•^	Nitric oxide
NPQ	Non-photochemical quenching
Nrf2	Nuclear factor erythroid 2–related factor 2
PAM	Peptidylglycine α-amidating monooxygenase
PDI	Protein disulfide isomerase
PGI	Phosphoglucose isomerase
PGM	Phosphoglucomutase
PMI	Mannose-6-phosphate isomerase
PMM	Phosphomannomutase
PSII	Photosystem II
RNS	Reactive nitrogen species
ROS	Reactive oxygen species
RSS	Reactive sulfur species
SOD	Superoxide dismutase
SQR	Sulfide:quinone oxidoreductase
SRB	Sulfate-reducing bacteria
TCEP	Tris(2-carboxyethyl)phosphine
TET	Ten-eleven translocation
TrxR	Thioredoxin reductase
UGDH	UDP-glucose dehydrogenase
UGPase	UDP-glucose pyrophosphorylase
VTC1	GDP-mannose pyrophosphorylase
VTC2	GDP-L-galactose phosphorylase
VTC4	L-Galactose-1-phosphate phosphatase
